# Low-density preference of the ambient and high-pressure polymorphs of dl-menthol

**DOI:** 10.1107/S2052252523002452

**Published:** 2023-04-21

**Authors:** Kinga Roszak, Andrzej Katrusiak

**Affiliations:** a Deparment of Material Chemistry, Adam Mickiewicz University, Uniwersytetu Poznańskiego 8, Poznań 61-614, Poland; ESRF, France

**Keywords:** polymorphism, compressibility, close-packing rule, polymorph prediction, molecular crystals, crystal engineering, intermolecular interactions

## Abstract

Lower-density polymorphs of dl-menthol were nucleated and crystallized in their high-pressure stability regions. Up to 0.30 GPa, the triclinic dl-menthol polymorph α, which is stable at atmospheric pressure, is less dense than a new β polymorph, which becomes stable above 0.40 GPa, but is less dense than the α polymorph at this pressure. The compression of polymorph α to at least 3.37 GPa is monotonic, with no signs of phase transitions. However, recrystallizations of dl-menthol above 0.40 GPa yield the β polymorph, which is less compressible and becomes less dense than α-dl-menthol. At 0.10 MPa, the melting point of the β polymorph is 14°C, much lower compared with those of α-dl-menthol (42–43°C) and l-menthol (36–38°C). The structures of both dl-menthol polymorphs α and β are very similar with respect to the lattice dimensions, the aggregation of OH⋯O molecules bonded into *C*_i_ symmetric chains, the presence of three symmetry-independent molecules (*Z*′ = 3), their sequence ABCC′B′A′, the disorder of the hydroxyl protons and the parallel arrangement of the chains. However, the different symmetries relating the chains constitute a high kinetic barrier for the solid–solid transition between polymorphs α and β, hence their crystallizations below or above 0.40 GPa, respectively, are required. In the structure of polymorph α, the directional OH⋯O bonds are shorter and the voids are larger compared with those in polymorph β, which leads to the reverse density relation of the polymorphs in their stability regions. This low-density preference reduces the Gibbs free-energy difference between the polymorphs: when polymorph α is compressed to above 0.40 GPa, the work component *p*Δ*V* counteracts the transition to the less dense polymorph β, and on reducing the pressure of polymorph β to below 0.40 GPa, its transition to the less dense polymorph α is also hampered by the work contribution.

## Abstract 2

The ambient-pressure dl-menthol polymorph α compressed above 0.40 GPa becomes metastable but more dense than the high-pressure polymorph β.

## 1. Introduction

The thermodynamic definition of pressure *p* = −∂*F*/∂*V*|*_T_* (Feynman, 1972 ▸), where *V* is the volume of any closed system, *T* its absolute temperature and *F* is the Helmholz free energy, can be rewritten in the form *p*∂*V* = −∂*F*. The assumption of positive work performed on the system (∂*F*) and the positive *p* value require that ∂*V* be negative. This implies the positive compressibility value β = −(1/*V*)(∂*V*/∂*p*) for any given homogenous phase within its stability region (*i.e.* for monotonic pressure-induced changes of the closed system of a specified chemical composition). Although it is not strictly forbidden by thermodynamic laws, the experimental evidence shows that the pressure-induced phase transitions also lead to either discontinuous or continuous volume reduction. The volume reduction is considered a necessary condition for the formation of new forms that are stable under high pressure (*e.g.* Bridgman, 1949 ▸; Nye, 1984 ▸; Newnham, 2005 ▸; Baughman *et al.*, 1998 ▸; Reichl, 1998 ▸), which is indeed often observed experimentally (*e.g.* Bridgman, 1964 ▸). Only very few exceptions indicate possible volume increases under high pressure: (i) when two polymorphs β and γ of 3-nitro­phenyl­disulfide were recrystallized at high temperature (Sobczak *et al.*, 2021 ▸); (ii) when the composition of the sample was increased due to the penetration of the pressure-transmitting liquid (Lee *et al.*, 2002 ▸; Li *et al.*, 2014 ▸) and gases; or (iii) when the compound underwent a chemical reaction under high pressure (Zhang *et al.*, 2020 ▸). Only the first of these exceptions (Sobczak *et al.*, 2021 ▸) fulfils the condition of the same chemical composition, although the higher-volume polymorphs, nucleated under high-pressure and high-temperature conditions, are metastable under normal conditions and are built of higher-energy conformers. Thus the volume increase was attributed to the increased internal energy and the entropy contributions to the Gibbs free energy, compensating the work contribution of increased volume under high pressure and temperature when these low-density polymorphs were nucleated. This mechanism involves transformations at metastable states and in this respect, it is analogous to the concept of negative compression through destabilization of metastable equilibria of constituents (Nicolaou & Motter, 2012 ▸).

**Figure 1:**
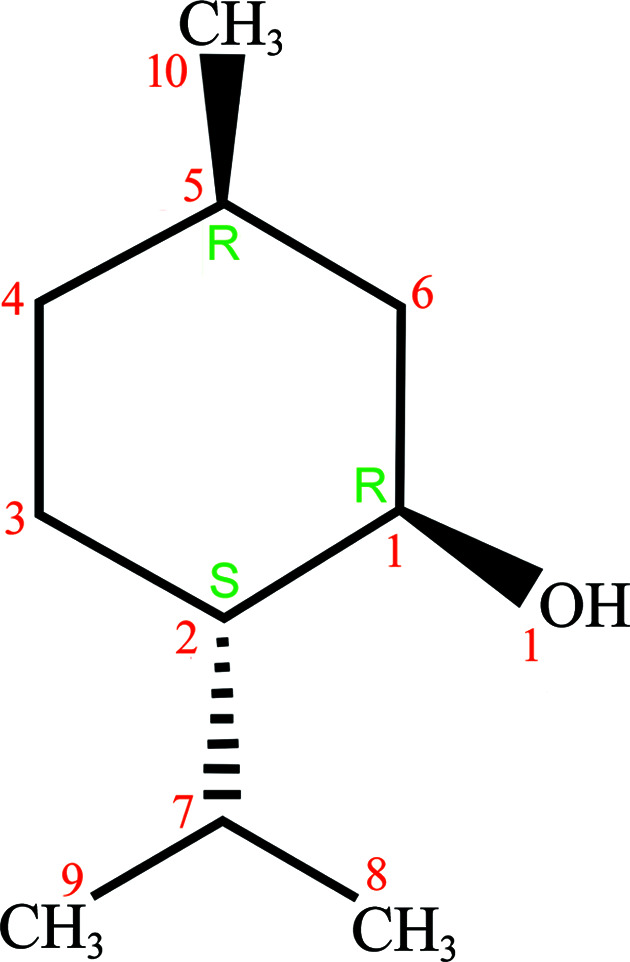
Chemical formula of l-(−)-menthol, its chiral centres (green) and atomic labels (red).

In the work described herein, we investigated a one-component compound, which does not change its composition, chemical formula or even the conformation of molecules, and we show that it favours the lower-density polymorphs under high-pressure conditions. To our knowledge, such a behaviour, when a low-density polymorph becomes stable under isothermally increased pressure, has not been reported to date. For this reason, as indicated above, it is often assumed that such a behaviour is not possible. Our case study on the well known fragrance compound dl-menthol demonstrates its preference to form the lower-density polymorphs, not only under atmospheric pressure, but also under high-pressure conditions. l-(−)-(1*R*,2*S*,5*R*)-menthol, naturally occurring in *Mentha piperita* and other *Mentha* species, is well known for its therapeutic qualities and as an ingredient in alimentary products and cosmetics (Fig. 1 ▸; Coleman *et al.*, 1998 ▸). Of the four concomitant l-menthol polymorphs, three of them are monotropic at atmospheric pressure, and their transformations were described over a century ago by Wright (1917 ▸). The fresh, sweet, mint, cooling and refreshing smell of naturally occurring l-(−)-menthol is similar to that of d-(+)-menthol, which is somewhat less minty and less refreshing, with bitter, phenolic and herbaceous notes. Hence the d-enantiomer and dl-racemate are used as substitutes for or complements to natural menthol (Bhatia *et al.*, 2008 ▸). The cooling sensation of l-(−)-menthol is about four times stronger than that of d-(+)-menthol, because the heat receptors in the skin are chiral (McKemy *et al.*, 2002 ▸; Bentley, 2006 ▸). Unlike l-(−)-menthol, the dl-racemate is mainly obtained by chemical synthesis and applied in ointments, cough drops and nasal inhalers, but also for flavouring food, cigarettes, liqueurs, cosmetics, perfumes *etc*. The solubility of methanol is most relevant to its applications: it hardly dissolves in water, 0.456 mg ml^−1^ at 25°C (298 K) (Yalkowsky *et al.*, 2010 ▸), but it is very soluble in ethanol, methanol (100 mg ml^−1^), di­ethyl ether, chloro­form petroleum ether, hexane; and it is freely soluble in glacial acetic acid; liquid petrolatum; and in mineral, fixed and volatile oils (Hopp & Lawrence, 2006 ▸; Sell, 1999 ▸; O’Neil, 2013 ▸).

Single-crystal X-ray diffraction (SCXRD) measurements revealed that l-(−)-menthol crystallizes in the trigonal space group *P*3_1_ (Ramsay & Rogers, 1952 ▸; Bombicz *et al.*, 1999 ▸) and dl-menthol is triclinic, with the space group *P* 1 (Corvis *et al.*, 2012 ▸). The binary phase diagram of the l- and d-enantiomers and the formation of the dl-menthol racemate compound were studied nearly five decades ago (Kuhnert-Brandstätter *et al.*, 1974 ▸) and later by Corvis *et al.* (2012 ▸; 2015 ▸), who also postulated new low-temperature metastable polymorphs of both l-(−)-menthol and dl-menthol at 233 K, but only the unit-cell dimensions and crystal symmetry (triclinic space group *P*1 for both), and no further structural information, were reported from the powder X-ray diffraction (PXRD) measurements. In our present study, we compressed and recrystallized dl-menthol under high-pressure in order to investigate its phase transitions and various possible forms, either polymorphs, solvates or even spontaneously separated enantiomers (Jacques *et al.*, 1981 ▸; Cai *et al.*, 2013 ▸; Marciniak *et al.*, 2014 ▸). We have obtained and characterized a new polymorph of dl-menthol, labelled polymorph β. It can be recovered under atmospheric pressure only at low temperature, because its melting point is about 14°C (287 K). The crystallographic parameters of our polymorph β are markedly different from those of the dl-menthol polymorph reported by Corvis *et al.* (2012 ▸, 2015 ▸). However, we found during our study that its most intriguing aspect is the counterintuitive density relation between the dl-menthol polymorphs. Between atmospheric pressure and 0.30 GPa, *i.e.* with the stability range of polymorph α extending to 0.40 GPa, its density is lower than the density of the new polymorph β, which is metastable in this pressure range. Above 0.30 GPa, in the stability region of polymorph β, its density becomes lower compared with that of polymorph α over-compressed to the same high-pressure value. To our knowledge, such stability regions of low-density high-pressure polymorphs are unprecedented in the literature and they are difficult to reconcile with the close-packing rule. The structural and thermodynamic analysis of this exceptional high-pressure behaviour of dl-menthol reveals new aspects of the close (Kitaigorodsky, 1973 ▸) and loose aggregation of molecules (Bujak *et al.*, 2008 ▸, 2018 ▸; Kaźmierczak & Katrusiak, 2013 ▸).

## 2. Experimental

**Figure 2:**
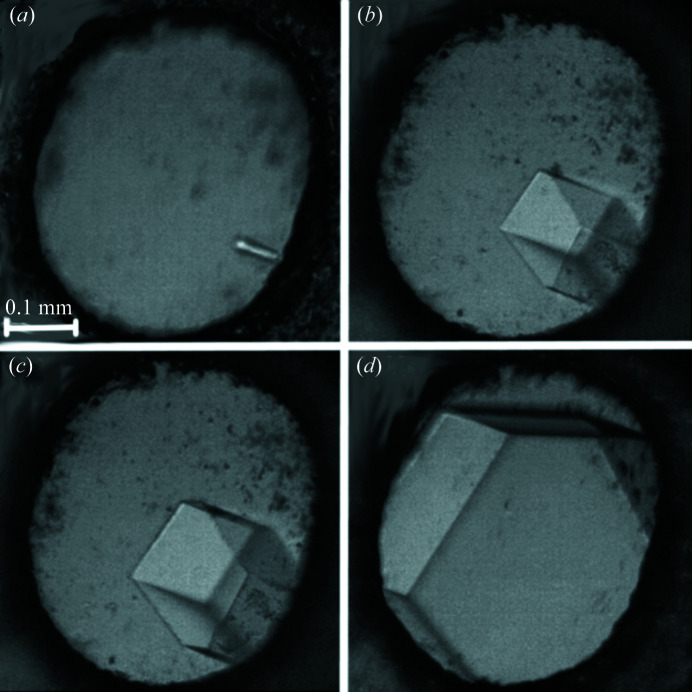
Single crystal of α-dl-menthol grown from the methanol:water mixture (1:2 *v*/*v*) under isochoric conditions in the DAC chamber at (*a*) 363 K, (*b*) 353 K, (*c*) 323 K, (*d*) 296 K and 0.10 GPa. Powder of crushed ruby chips for pressure calibration is scattered close to the gasket edge.

High-pressure experiments were performed in a Merrill–Bassett diamond anvil-cell (DAC, Merrill & Bassett, 1974 ▸), modified by mounting the anvils directly on steel backing plates with conical windows (Katrusiak, 2008 ▸). Crystals of dl-menthol were investigated either by gradually compressing a single crystal grown at 294 (2) K and atmospheric pressure, or by high-pressure recrystallizations and growing single crystals under isothermal and isochoric conditions from the solutions in dry methanol, ethanol, methanol:water mixture (1:2 *v*/*v*) and ethanol:water mixture (2:1 *v*/*v*), as shown in Figs. 2 ▸ and S1–S5 of the supporting information.

For the isothermal recrystallizations, the concentration of dl-menthol was adjusted to nucleate the crystal either below or above 0.40 GPa in order to check the possible role of increased temperature and entropy changes for the formation of polymorph β. Also, isochoric recrystallizations from pure molten dl-menthol were performed (Fig. S6) to eliminate the possibility of stoichiometric or stochastic cocrystallization of dl-menthol with solvent molecules. The isochoric recrystallizations of pure dl-menthol required 453 K for melting the sample at 0.90 GPa and 483 K for melting the sample at 1.28 GPa. We also performed seeded crystallizations above 0.40 GPa, when a small seed of polymorph α was left, when the temperature was lowered, but these recrystallizations also resulted in the crystals of polymorph β.

**Figure 3:**
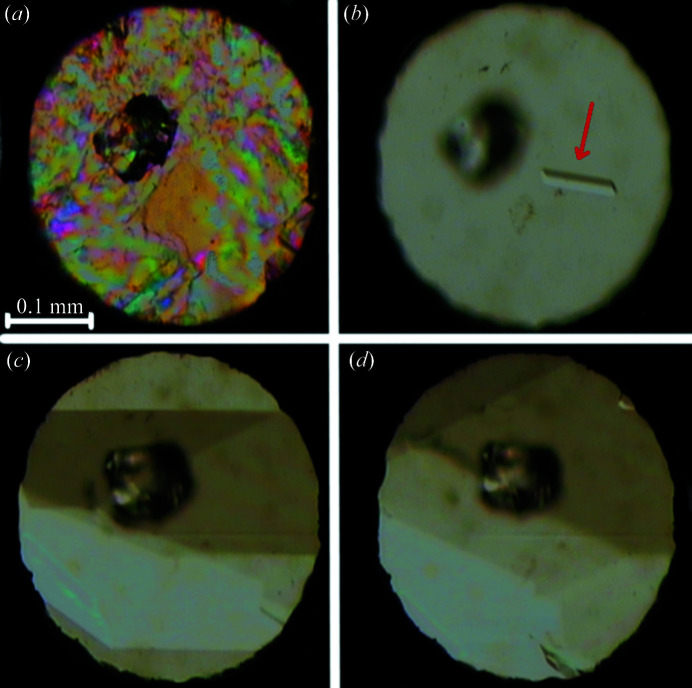
Single crystal of β-dl-menthol grown from the ethanol:water mixture (2:1 *v*/*v*) under isochoric conditions in the DAC chamber: (*a*) spontaneous powder precipitation at 0.64 GPa viewed under polarized light, (*b*) one seed (indicated by the red arrow) at 365 K, (*c*) 323 K and (*d*) 0.64 GPa and 296 K. A large ruby chip for pressure calibration lies close to the centre of the chamber.

The structures of high-pressure polymorphs α and β were determined by SCXRD up to 3.37 and 2.90 GPa, respectively, although we plotted the data up to 2.5 GPa only to better represent the region around 0.40 GPa. The compression of dl-menthol crystals was determined for the sample grown under ambient conditions and then mounted in the DAC. The gaskets were made of 0.20 mm-thick Inconel foil and the initial diameter of the spark-eroded holes was 0.45 mm. Glycerine was used as the hydro­static medium. Pressure in the DAC chamber was calibrated by the ruby-fluorescence method (Piermarini *et al.*, 1975 ▸; Mao *et al.*, 1986 ▸) with a Photon Control Inc. spectrometer, affording an accuracy of 0.02 GPa; the calibration was repeated before and after each diffraction measurement. Above 0.40 GPa the isochoric recrystallizations of molten dl-menthol and the solutions in methanol:water and ethanol:water mixtures (2:1 *v*/*v*) yielded a new monoclinic polymorph β (Fig. 3 ▸). Such alcohol:water mixtures start to separate under high pressure above 1 GPa, as ice VI crystallizes, which increases the methanol and ethanol concentration; eventually they become nearly pure and remain hydro­static up to their freezing pressures at 3.50 and 1.80 GPa (Allan *et al.*, 1998 ▸; Allan & Clark, 1999 ▸), respectively. The hydro­static limit of glycerine was assessed as 3.00 GPa (Hazen & Finger, 1982 ▸). The crystallizations of dl-menthol from its melt were undertaken in order to exclude the possibility of stochastic solvation (Roszak & Katrusiak, 2021 ▸; Olejniczak *et al.*, 2022*a* ▸), while the non-hydro­static conditions due to the thermal contraction were ignored despite the markedly non-isotropic thermal contraction of the β-dl-menthol crystal (Figs. S7–S10).

Low-temperature SCXRD data were measured on Oxford Diffraction diffractometers Xcalibur and SuperNova equip­ped with low-temperature Cryosystem attachments. High-pressure SCXRD data were recorded on diffractometers KUMA KM4-CCD and Xcalibur, equipped with EOS-CCD detectors, according to the procedure described previously (Budzianowski & Katrusiak, 2004 ▸). The *CrysAlisPro* software (CrysAlisPro Rigaku Oxford Diffraction, 2015 ▸) was used for collecting diffraction data and their preliminary reduction. The sample reflections overlapping with diamond reflections were eliminated, and corrections for the DAC and sample absorption and for the beam shadowing by the gasket were applied. *Olex2* (Dolomanov *et al.*, 2009 ▸) was used, the crystal structures were solved by direct methods within *SHELXT* (Sheldrick, 2015*b* ▸) and refined by least-squares using *SHELXL* (Sheldrick, 2015*a* ▸). Anisotropic displacement parameters (ADPs) were applied for non-hydrogen atoms in all low-temperature structures, whereas for the ambient-conditions and high-pressure experiments ISOR constraints were applied for all carbon and oxygen atoms (*cf. SHELXL* input instructions in the supporting CIFs). The hydrogen atoms were located from the molecular geometry and their isotropic ADP values *U*_iso_ were constrained to 1.2*U*_eq_ of their carriers. In dl-menthol, the starting model with the hydroxyl proton disordered was assumed. The hydroxyl hydrogen atoms were refined with geometric constraints (OH distance 0.81 Å) and were assigned *U*_iso_ = 1.2*U*_eq_ of the oxygen atoms. We investigated the disorder of hydroxyl hydrogen atoms (labelled H1) by lowering the symmetry to space group *P*1 and refining the site occupation factors as free variables for two possible sites for each of the six independent hydrogen atoms. Their site occupation factors (SOFs) were refined as free variables with the constraints SOF(H1*i*1) + SOF(H1*i*2) = 1, where *i* = A, B, C for three independent molecules and the last digits of the labels, 1 and 2, denote the first and second of two sites of the disordered hydroxyl hydrogen atom. In all experiments performed between 296 and 100 K, the SOFs refined to values close to 0.5 and therefore we assumed that all three hydroxyl protons are 50:50 disordered. After removing the hydroxyl protons, in each OH⋯O hydrogen bond two similar electron-density peaks corresponding to the disordered hydrogen sites were found in the difference Fourier maps. Thus, no reasons were found for rejecting the symmetry of space group *P* 1, which implies the 50:50 disorder of hydroxyl hydrogen atoms in the final refinements. Structural drawings were prepared using the program *Mercury* (version 4.0; Macrae *et al.*, 2020 ▸).

High-pressure PXRD measurements were performed for the sample compressed in the DAC, according to the procedure described by Skumiel (2010 ▸). The ethanol:water mixture (1:3 *v*/*v*) was used as the solvent and hydro­static fluid. The Xcalibur diffractometer equipped with an 0.3 mm collimator was used, and the diffraction images were recorded for the sample rotated by ±15° about the ω axis from its 0° position, for the χ axis at 0, 30, 60 and 90° positions. The diamond reflections were eliminated, the background recorded beforehand for the empty DAC was subtracted and the residue intensity was integrated at constant 2θ angles. Before each measurement the sample was almost completely dissolved by heating and then left to crystallize under isochoric conditions up to 2.23 GPa (*cf*. Fig. S11). The crystallographic information has been deposited in the Cambridge Structural Database (Groom *et. al.*, 2016 ▸) with deposition nos. 2206626–2206658. Selected crystal data are summarized in Table 1 ▸. Experimental and crystallographic details are listed in Tables S1–S2 of the supporting information.

Differential scanning calorimetry (DSC) measurements were performed for the samples sealed in aluminium capsules in N_2_ atmospheres on a Setsys 1200 Setaram instrument between 140 and 573 K at a scan speed of 5 K min^−1^ (*cf*. Fig. S12).

## 3. Results and discussion

**Figure 4:**
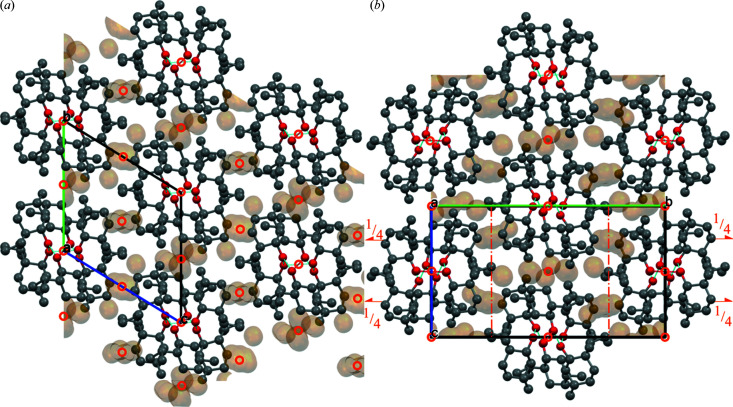
Structures of (*a*) α-dl-menthol and (*b*) β-dl-menthol projected down the OH⋯O bonded chains. The hydrogen atoms have been omitted for clarity. The voids are represented as contact surfaces for the probing radius 0.85 and step 0.1 Å (*cf*. Fig. S15). The symmetry elements of space groups *P* 1 (*a*) and *P*2_1_/*n* (*b*) are marked in orange.

Under normal conditions (273 K and 1006 hPa), dl-menthol forms triclinic crystals, hereafter referred to as polymorph α (α-dl-menthol, Table 1 ▸; Figs. 4 ▸, S13 and S14). The crystals of α-dl-menthol are built from ⋯OH⋯OH⋯ bonded chains. In the chains, three independent molecules (*Z*′ = 3), denoted A, B and C, are arranged in the sequence [ABCCBA]_*n*_. The ABC intervals are of the same chirality, reversed by two non-equivalent inversion centres located between molecules AA and CC. All hydroxyl atoms (H1) are disordered 50:50 across two sites in OH⋯O hydrogen bonds (*cf*. Experimental) and the DSC (*cf*. Fig. S12) of the α-dl-menthol crystals revealed no anomalous heat flow down to 140 K. This result is consistent with our low-temperature X-ray diffraction measurements on the α-dl-menthol crystals showing the persistence of the disorder of hydroxyl hydrogen atoms down to 120 K at least, and no new phases, in particular no metastable triclinic dl-menthol postulated at 233 K by Corvis *et al.* (2012 ▸); its occurrence was excluded within the conditions of our experiments. Interestingly, the similar feature of the same number of three independent molecules (*Z*′ = 3) is also characteristic of the l-menthol enantiomorph, although the structure of helices of OH⋯O bonded l-menthol molecules is different from that of OH⋯O bonded dl-menthol chains.

**Figure 5:**
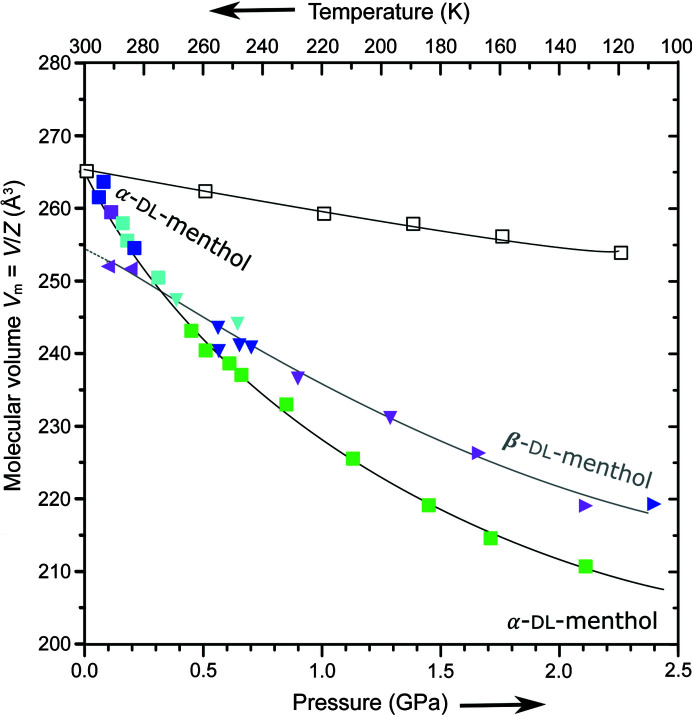
Pressure (full symbols) and temperature (open squares) dependence of molecular volume (*V*_m_ = *V*/*Z*) for α-dl-menthol (squares) obtained at atmospheric pressure (green squares); and recrystallized up to 0.40 GPa from methanol:water solution (blue), ethanol:water solution (cyan) as well as from the pure melt (violet); all recrystallizations above 0.40 GPa yielded β-dl-menthol (triangles down), which also persisted after further compression (triangles right) and decompression below 0.40 GPa (triangles left). All estimated standard deviations are smaller than the plotted symbols. The least-squares functions (plotted lines) fitted to the experimental points are *V*_α_(*p*) = 265.0 (4) − 52 (1)*p* + 18 (2)*p* ^2^ − 2.5 (6)*p* ^3^ and *V*_β_(*p*) = 255 (2) − 21 (6)*p* − 1(7)*p* ^2^ + 1(2)*p* ^3^ (Å^3^).

We found that the α-dl-menthol crystals, obtained and mounted in the DAC under ambient conditions in our laboratory, can be compressed with no signs of anomalies to 2.20 GPa at least (Fig. 5 ▸). The smooth compression of the crystal volume and unit-cell parameters are indications that the crystal remains in the same phase, whereas anomalous compression (discontinuities or changed rate of compression) would indicate phase transitions to other phases. Generally, the absence of detectable anomalies cannot be directly connected with the thermodynamic stability of crystalline or liquid phases, because of the possible effects of supercompressing (Katrusiak *et al.*, 2011 ▸; Paliwoda *et al.*, 2012 ▸; Anioła & Katrusiak, 2015 ▸; Roszak *et al.*, 2016 ▸; Fedorov *et al.*, 2017 ▸; Andrzejewski & Katrusiak, 2017 ▸; Sobczak & Katrusiak, 2017 ▸; Safari & Katrusiak, 2021 ▸; Olejniczak *et al.*, 2022*b* ▸) or supercooling (Weineerg, 1908 ▸) the phases.

**Figure 6:**
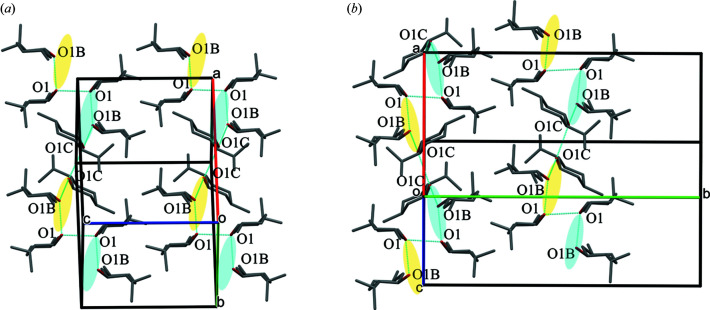
Projections of neighbouring OH⋯OH bonded chains along the direction perpendicular to their axes in (*a*) α-dl-menthol and (*b*) β-dl-menthol. Chain intervals ABC of l-(−)(1*R*,2*S*,5*R*)-menthol molecules are highlighted in blue and of d-(+)(1*S*,2*R*,5*S*)-menthol molecules are highlighted in yellow. The dashed ovals indicate analogous regions of the shortest contacts between chains. Hydrogen atoms have been omitted for clarity.

In another series of experiments, when the single crystals of dl-menthol were recrystallized from the water:ethanol mixture under pressure in the DAC, the same triclinic polymorph α was obtained up to 0.40 GPa. Above 0.40 GPa, a new monoclinic form, hereafter denoted β-dl-menthol, was crystallized (Table 1 ▸). This new polymorph β-dl-menthol could not be obtained by compressing the ambient-pressure polymorph α up to 3.37 GPa. Such high-pressure polymorphs, accessible via *in situ* recrystallization under pressure, were found for imidazole (Paliwoda *et al.*, 2012 ▸), 4,4′-bipiridium hydro­bromide monohydrate (Anioła & Katrusiak, 2015 ▸), 3-hy­droxy-4,5-di­methyl-1-phenyl­pyridazin-6-one (Roszak *et al.*, 2016 ▸), di-*p*-tolyl disulfide (Sobczak & Katrusiak, 2017 ▸), resorcinol (Safari & Katrusiak, 2021 ▸) and other compounds (Fedorov *et al.*, 2017 ▸; Andrzejewski & Katrusiak, 2017 ▸; Olejniczak *et al.*, 2022*b* ▸). However, the molecular volume of those polymorphs accessible through recrystallization is smaller compared with the compressed ambient-pressure polymorph. Our experiments show that the volume of β-dl-menthol is larger compared with α-dl-menthol and this difference increases with increasing pressure (Fig. 5 ▸). This was a surprising result because, in general, one expects the high-pressure polymorphs to be more dense compared with the low-pressure polymorphs, when both polymorphs are compared under the same pressure conditions. Moreover, the close similarity between the structures of α-dl-menthol and β-dl-menthol could imply their similar compressibility. Fig. 4 ▸ shows that both these structures are built of similar chains of OH⋯O bonded molecules. These chains are nearly identical in shape (Figs. 6 ▸, S13 and S14): each contains three independent molecules in sequence A′ABCC′′, with the inversion centres between the A′A molecules and between the CC′′ molecules (in polymorph β, the prime indicates the molecule transformed through symmetry code *x* , *y* , 1 − *z* and bis denotes 1 − *x*, *y* , 1 − *x*; in polymorph α, the prime denotes the symmetry code 1 − *x*, 2 − *y*, *z* and bis denotes 2 − *x*, 2 − *y*, *z* ).

It appears that when viewing the structure along the chains (*i.e.* along **a** in polymorphs α and β), the arrangements of chains are nearly identical (Figs. 4 ▸ and 6 ▸) and their shortest contacts to neighbouring chains are also very similar. However, the chains are shifted differently along their axes with respect to the six close chains: in polymorph α translated along ±*b*_α_, ±[011]_α_, ±*c*_α_; and in polymorph β translated along ±*c*_β_, or transformed by glide plane *n* and screw axis 2_1_, to the positions along ±[011]_β_ and ±[011]_β_, as illustrated in Figs. 4 ▸, S7 and S8.

Furthermore, a striking similarity connects the lattices of both polymorphs: the lattice vectors of β-dl-menthol can be transformed to approximate those of α-dl-menthol through the matrix equation

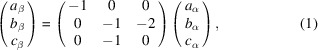

where subscript β refers to the monoclinic β-dl-menthol and subscript α denotes the triclinic α-dl-menthol. The reverse matrix transforms the lattice of polymorph β into that of polymorph α

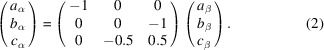

**Figure 7:**
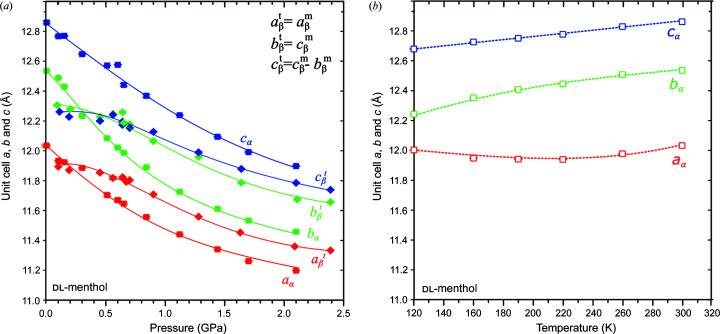
(*a*) Pressure and (*b*) temperature dependence of unit-cell parameters *a*, *b*, *c* in α-dl-menthol (squares and α subscripts) and β-dl-menthol (diamonds and β subscripts; *cf*. Fig. S7). For convenient comparisons of analogous lattice directions, the unit-cell parameters of the monoclinic β-dl-menthol (superscript *m* in the legend) are transformed to the triclinic lattice [superscript t, *cf.* equation (2)]. The pressure dependence of the unit-cell parameters for the lattices in the settings of polymorphs α and β are plotted in Figs. S7–S9.

According to Equation (1), the triclinic unit cell of α-dl-menthol at 0.1 MPa can be represented as the following pseudo-monoclinic unit cell: *a* ^m^ = 12.035 Å, *b* ^m^ = 22.809 Å, *c* ^m^ = 12.536 Å, α^m^ = 88.40°, β^m^ = 103.93° and γ^m^ = 107.52° [superscript m indicates the unit-cell dimensions of polymorph α transformed according to equation (1) to the monoclinic setting of polymorph β]. The unit-cell parameters of α-dl-menthol at 0.60 GPa transformed in this way to give *a* ^m^ = 11.581 Å, *b* ^m^ = 22.336 Å, *c* ^m^ = 12.222 Å, α^m^ = 88.57°, β^m^ = 103.76° and γ^m^ = 108.44°. Both these sets of pseudo-monoclinic unit cells are similar to the unit cell of β-dl-menthol (Table 1 ▸), except that angle γ significantly diverges from 90°. This departure from 90° increases with pressure, by over 1.0° GPa^−1^, which shows that the compressed lattices of polymorphs α and β become increasingly different (Fig. S9). Equations (1) and (2) can be used for convenient comparions of the dimensions and compressions of the structures of polymorphs α and β in their corresponding directions. Fig. 7 ▸ shows that the parameters *a* ^t^ and *b* ^t^ of the triclinic lattice are similar in polymorphs α and β, whereas the *c* ^t^ parameters differ by about 0.6 Å. These smaller differences occur along the OH⋯O bonded chains (parameter *a* ^t^) and in this direction between the chains where they are related by inversion centres (parameter *b* ^t^), whereas the largest difference along *c* ^t^ is between the chains related by different symmetry elements: the inversion centres in polymorph α, and the glide planes *n* or screw axes 2_1_ in polymorph β (Fig. 4 ▸). The strongest difference in compression occurs up to about 0.5 GPa, when polymorph α, owing to the presence of voids supported by directional OH⋯O bonds (Fig. S15), is much softer than polymorph β. Above 1.0 GPa the compressions along *a* ^t^ and *b* ^t^ become similar, but the compression along *c* ^t^ continues to be considerably lower for polymorph β (Fig. 7 ▸). The crystal-strain analyses for compressed polymorphs α and β are presented in Tables S3, S4 and Fig. S10.

The α-dl-menthol polymorph is highly compressible, considerably more so than most crystals of organic compounds, and at 1.00 GPa its volume is reduced by about 13.2%. It is connected with an exceptionally low density of α-dl-menthol (Table 1 ▸), due to the presence of many small voids constituting nearly 25% of the structure at 0.10 MPa, according to the probing-sphere calculations (with a probing-sphere radius of 0.60 Å and a step of 0.10 Å, *cf.* Fig. S14). The compressibility of α-dl-menthol is strongly non-linear (Figs. 5 ▸ and S17), which is characteristic of molecular crystals with large volumes of voids. The compressibility of α-dl-menthol at 0.10 MPa is β_α_(0.1 MPa) = 0.198 GPa^−1^, compared with the nearly five times smaller compressibility at 2.00 GPa, β_α_(2.0 GPa) = 0.0415 GPa^−1^. The compressibility of β-dl-menthol at 0.10 MPa is estimated to be β_β_(0.1 MPa) = 0.084 GPa^−1^ compared with the more than four times smaller compressibility at 2.00 GPa, β_β_ (2.0 GPa) = 0.018 GPa^−1^.

The very high compressibility of polymorph α-dl-menthol at 0.10 MPa (Fig. 5 ▸) is two and half times larger than that of β-dl-menthol (see the compressibility values above); at 0.40 GPa the compressibility of polymorph α is still double that of polymorph β: 0.149 compared with 0.077 GPa^−1^, respectively. The smaller compressibility of polymorph β compared with that of polymorph α (Fig. 5 ▸) can be connected to initially significantly smaller intermolecular voids in polymorph β (Fig. S15). Consequently, starting from about 0.30 GPa, the molecular volume of the low-pressure polymorph α becomes smaller than that of the high-pressure polymorph β. We have double checked this counterintuitive result by performing the recrystallizations as a function of pressure in the DAC from different solvents, dry methanol, ethanol and finally from the molten pure dl-menthol. All these experiments confirmed that, up to 0.30 GPa, the density of polymorph α is lower than that of polymorph β and above 0.30 GPa the density of polymorph β becomes lower than that of polymorph α. Due to the molecular volume differences, the Gibbs free energy difference between the stable and unstable polymorphs is reduced by the energy value 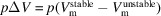 , which acts against the stability of the polymorphs obtained by recrystallization: for polymorph α up to 0.30 GPa, this work contribution 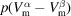 is positive; and above 0.40 GPa in the stability region of polymorph β, the product 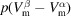 is also positive (*cf*. Figs. 5 ▸ and 8 ▸). Moreover, the recrystallizations of the pure molten dl-menthol yielded polymorph α below 0.40 GPa and polymorph β above 0.40 GPa. Because no other compounds except dl-menthol were present in the DAC chamber, this series of experiments showed that no solvent effects, such as stochastic sorption, take place (Roszak & Katrusiak, 2021 ▸; Olejniczak *et al.*, 2022*a* ▸).

**Figure 8:**
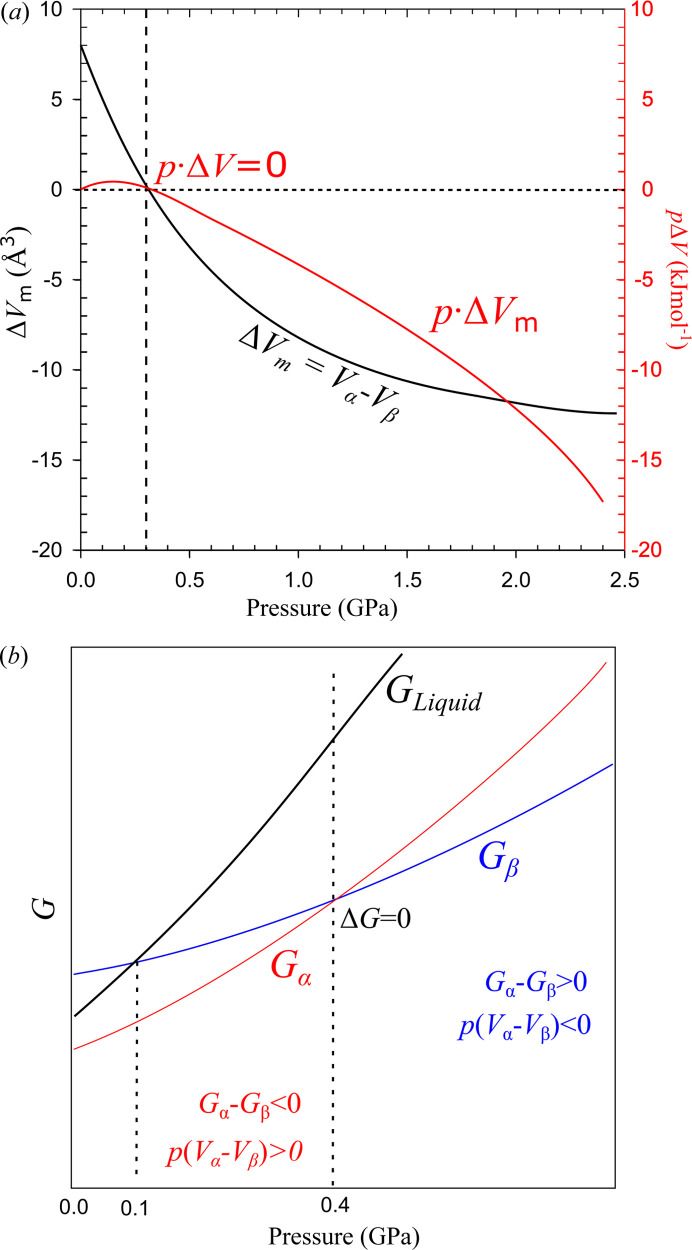
(*a*) Molecular volume difference Δ*V* between dl-menthol polymorphs α and β and the work contribution *p*Δ*V* to the Gibbs free energy *G*; (*b*) plots of Gibbs free-energy functions *G*_Liquid_, *G*_α_ and *G*_β_ as a function of pressure, the dotted lines indicate the experimentally observed melting pressure of polymorph β at 0.1 GPa and 296 K, and the point of changed preference for the crystallization between polymorphs α and β at 0.4 GPa. Note the volume difference Δ*V* = 0 (and hence Δ*Vp* = 0) at *p* = 0.30 GPa in (*a*) occurs at a lower pressure than Δ*G* = 0 at *p* = 0.40 GPa in (*b*).

The larger volume and hence lower density of the high-pressure polymorph is difficult to reconcile with the typical behaviour of one-component compounds, which undergo a pressure-induced phase transition. However, no phase transition has been observed in any of our high-pressure experiments on dl-menthol, even when the samples were kept at high pressure for several months. The polymorphs α and β were obtained solely by recrystallizations. Thus, their molecular volumes can be regarded as independent parameters, like the Gibbs free energy functions *G*_α_ and *G*_β_ of the polymorphs, and

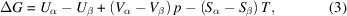

where the internal energy *U* and entropy *S* of polymorphs, α and β, depend on pressure *p* and temperature *T*. The high-pressure recrystallizations and SCXRD studies on dl-menthol show that the zero value of Δ*G* = 0 occurs at 0.40 GPa, close to the pressure when Δ*V* = 0 (Fig. 8 ▸). If we assume the same pressure for Δ*G* = 0 and Δ*V* = 0, equation (3) simplifies to

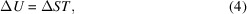

where Δ*U* = *U*_α_ − *U*_β_ and Δ*S* = *S*_α_ − *S*_β_. Fig. 8 ▸ shows that, between 0.10 MPa and 0.40 GPa, the work contribution considered for transforming the metastable β polymorph to the stable α polymorph *p*Δ*V* = *p*(*V*_α_ − *V*_β_) is positive, and thus it acts against such a transition. However, the *p*(*V*_α_ − *V*_β_) value is smaller than 0.5 kJ mol^−1^, which can be overcome by the differences in the internal energy (Δ*U*) and entropy (Δ*S* *T*) contributions, usually much higher for isomers and polymorphs of molecular hydrogen-bonded crystals (Madsen & Larsen, 2007 ▸; Kofoed *et al.*, 2019 ▸). Above 0.40 GPa, when the β polymorph becomes stable, the work contribution *p*Δ*V* = *p*(*V*_β_ − *V*_α_) considered for transforming the metastable α polymorph to the stable β polymorph is positive again and hence it acts against the transition again. Despite this larger positive work contribution (Fig. 8 ▸), the β polymorph nonetheless becomes more stable than the α polymorph due to the Δ*U* and Δ*S* *T* contributions. The comparison of the compressed structures of polymorphs α and β reveals systematic significant structural differences connected to the internal energy. The compression of all parameters is initially much stronger for polymorph α. The compression of parameters *a*_α_ and *a*_β_ along the OH⋯O bonded chains correlates with the compression of the hydrogen bonds (Figs. 6 ▸ and 9 ▸). It is plausible that the stronger compression of the OH⋯O bonds in polymorph α significantly increases the internal energy and reduces the entropy connected with the atomic displacement parameters, which counteract the work contribution and destabilize the α-dl-menthol structure above 0.40 GPa. The stronger compression of OH⋯O bonds in polymorph α contrasts with the stronger compression of van der Waals contacts H⋯H in polymorph β, plotted in Figs. S17 and S18.

The significantly larger voids in α-dl-menthol up to about 0.50 GPa are consistent with the lower density of this polymorph up to about 0.40 GPa (Fig. S15). The similar volumes of voids in α- and β-dl-menthol above 0.50 GPa can be regarded as an indication that the lengths of chains (*a*_β_ > *a*_α_) contribute most to the lower density of β-dl-menthol above 0.40 GPa (Figs. 7 ▸, S9 and S15).

**Figure 9:**
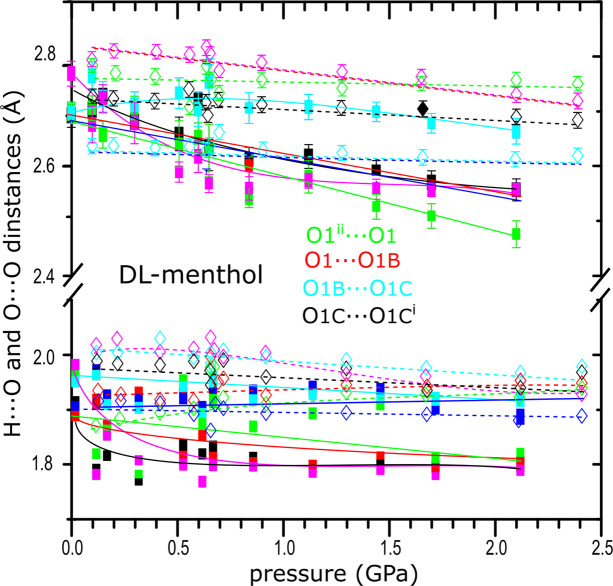
Pressure dependence of H⋯O and O⋯O distances in four independent hydrogen bonds (see legend) in dl-menthol polymorphs α (full squares, full lines) and β (open diamonds, dashed lines). The temperature dependence of these dimensions as well as the pressure-induced changes in OH⋯O angles are plotted in Figs. S19 and S23.

In the structure of polymorph α three of four independent OH⋯O hydrogen bonds are monotonically compressed, whereas one hydrogen bond initially increases in length, which is clearly seen for the O1B⋯O1C distance, and above about 1.0 GPa it is compressed at a slower rate compared with other hydrogen bonds (Fig. 9 ▸). The OH⋯O angle undergoes a large change, from 148 to 175° in the pressure range from 0.10 MPa to 0.10 GPa (Fig. S19). Above about 0.50 GPa, all the OH⋯O angles decrease with pressure and the smallest of the angles becomes close to 130° at about 1.50 GPa. It is also characteristic that on average all OH⋯O hydrogen bonds in polymorph α are *ca* 0.10 Å shorter than the corresponding four independent hydrogen bonds in polymorph β and this average difference in length does not change significantly with pressure. It was postulated that the bulky molecules cannot get close enough to form short hydrogen bonds OH⋯O owing to steric hindrances interfering with the symmetry of glide planes and 2_1_ screw axis (Brock & Duncan, 1994 ▸), which is in agreement with the structures of dl-menthol polymorphs. The longer OH⋯O bonds in β-dl-menthol correspond with the longer chains in this polymorph and with the unit-cell dimension *a*_β_ > *a*_α_. The compression of crystals is also reflected in conformational changes of molecules, up to about 20° in torsion angles, changing their values between the polymorphs and as a function of pressure (Figs. S20–S22).

In general, experimental evidence shows there are numerous compounds that, under ambient conditions, form stable polymorphs less dense than the more dense unstable polymorphs, which are either concomitant or can be obtained under high pressure (*e.g.* Boldyreva *et al.*, 2002 ▸; Krawczyk & Gdaniec, 2005 ▸; Nelyubina *et al.*, 2010 ▸; Adhikari *et al.*, 2015 ▸; Zieliński & Katrusiak, 2013 ▸; Safari & Katrusiak, 2021 ▸). However, in all these cases, the high-density metastable polymorph continues to be more dense in its high-pressure stability region. To our knowledge, the dl-menthol polymorphs α and β document for the first time the existence of a compound, for which the high-pressure polymorph (β), obtained under isothermal conditions, becomes less dense than the low-pressure polymorph (α). These polymorphs can coexist and below 0.40 GPa the less dense polymorph α is stable, whereas at pressures above 0.40 GPa polymorph α becomes more dense and less stable than polymorph β, despite the fact that no chemical, compositional or even a significant conformational change (Fig. S24) take places. This effect of high-pressure favouring the less dense polymorph of dl-menthol is similarly as counterintuitive as the effect of increased chemical composition reducing the molecular volume. It is expected that new components added to a structure will increase its volume; however, there are rare observations that the molecular volumes of anhydrates are smaller than those of their hydrates (Zieliński & Katrusiak, 2015 ▸; Andrzejewski *et al.*, 2017 ▸).

In order to confirm that polymorph β is stable at the same temperature as polymorph α, at 296 K, we performed isothermal crystallizations from dl-menthol dissolved in iso­propanol, and nucleation occurred above 0.40 GPa. These isothermal *in situ* recrystallizations yielded the β polymorph, identified by PXRD, as exemplified in Fig. S25.

**Figure 10:**
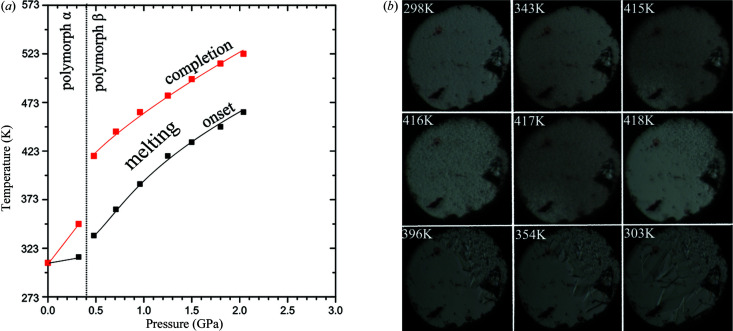
(*a*) Melting temperature of pure α-dl-menthol and β-dl-menthol compressed and recrystallized in the DAC chamber: the black squares mark the onset of melting of the crystal filling the volume of the chamber, and the red squares mark the melting completion, according to the microscopic observation of the sample, as exemplified for the heating run of the sample at the starting pressure 0.48 GPa in (*b*). The increased temperature for the full melting (red squares) compared with that at the start (black squares) results from the pressure–temperature interdependence for the isochoric process: the larger portion of the sample is molten, the higher pressure is in the DAC chamber. Due to the thermal expansion of the sample, the pressure measurements at 296 K plotted in (*a*) are lower than the actual values for which the melting onset, and even more so the melting completion, took place. In the DAC chamber several small irregular ruby chips are placed close to the gasket walls for the ruby-fluorescence pressure measurements.

We also considered the possibility that polymorph β-dl-menthol is metastable in a wide range of pressures, even above 0.40 GPa. The formation of the metastable β polymorph would be consistent with Ostwald’s rule of stages. In such a case, the metastable phase would have a lower melting point than the stable phase, not only below 0.40 GPa, but also above this pressure. Hence, in another series of experiments we gradually compressed the pure dl-menthol sample in the DAC and for each pressure point we recorded the temperature of the beginning and of the full melting of the sample (Fig. 10 ▸), determined by microscopic observations. The temperature required for melting the sample in the DAC chamber drastically increases above 0.40 GPa. This increase in temperature of about 80 K above 0.40 GPa, coinciding with the formation of polymorph β, indicates that it becomes more stable than polymorph α, which implies that polymorph β is not metastable above 0.40 GPa.

## 4. Conclusions

According to the close-packing rule for molecular crystals (Kitaigorodsky, 1973 ▸), their dense structures should be favoured under specific thermodynamic conditions. We have shown that this is not the case for dl-menthol polymorphs α and β when nucleated and crystallized in their low- and high-pressure stability regions, respectively. As expected, up to 0.40 GPa, the triclinic dl-menthol polymorph α, stable at atmospheric pressure, is less dense than the high-pressure β polymorph revealed here. But the exceptional observation is that the high-pressure polymorph β-dl-menthol is less dense in its stability region above 0.40 GPa than the super-compressed ambient-pressure polymorph α. We have rationalized this counterintuitive pressure–density relation between the dl-menthol polymorphs by the shorter OH⋯O bonds enforcing larger voids in polymorph α compared with the considerably longer OH⋯O bonds allowing a more compact structure of polymorph β. Like in the structure of H_2_O ice *I*_h_, in dl-menthol polymorph α the OH⋯O bonds dominate the cohesion forces, achieved at the cost of close-packing of the molecules. This loose packing in dl-menthol polymorph α results in its high compressibility, which in turn leads above 0.30 GPa to its higher-density compared with the less-compressible polymorph β. Therefore, the densities of dl-menthol polymorphs α and β in their stability regions below and above 0.40 GPa, respectively, are lower compared with the densities of their metastable counterparts. These density relations are essential for the thermodynamic properties of the α and β polymorphs, because the work contribution *p*Δ*V* to the Gibbs free energy favours the unstable polymorph. It reduces the energy difference between the Gibbs free energies of the polymorphs, |*G*_α_ − *G*_β_|*_T_*, while the kinetic barrier for the transition between the polymorphs, requiring considerable structural rearrangements, remains high. Consequently, the kinetics of the possible transitions between the polymorphs are very slow. Indeed, we observed no solid-state phase transitions from polymorph α to polymorph β when the pressure was increased to above 0.40 GPa, and from polymorph β to polymorph α when pressure was released to below 0.40 GPa. For obtaining the other polymorph under high pressure, the dl-menthol sample had to be either molten or dissolved. The α- and β-dl-menthol polymorphs exemplify an extremely rare system, for which the high-pressure polymorph is less dense compared with the metastable (super-compressed) one. Such a phenomenon, although counterintuitive and unprecedented in the literature, is not forbidden by any thermodynamic law, and therefore it widens our understanding of the general properties of molecular crystals. dl-Menthol exemplifies a chemical compound, which even under high pressure is not stable in its most dense polymorphs. This observation can be used for the interpretation of theoretically predicted models of crystal structures, where the density and internal energy criteria are generally applied (Bhardwaj *et al.*, 2019 ▸; Tchoń *et al.*, 2021 ▸; Price & Price, 2022 ▸).

## 5. Related literature

The following references are cited in the supporting information: Cliffe & Goodwin (2012 ▸); Langreiter & Kahlenberg (2015 ▸).

## Supplementary Material

Crystal structure: contains datablock(s) alpha_DL_0.01MPa, alpha_DL_0.10GPa, alpha_DL_0.15GPa, alpha_DL_0.30GPa, alpha_DL_0.51GPa, alpha_DL_0.60GPa, alpha_DL_0.65GPa, alpha_DL_0.84GPa, alpha_DL_1.12GPa, alpha_DL_1.44GPa, alpha_DL_1.70GPa, alpha_DL_2.10GPa, alpha_DL_3.37GPa, alpha_DL_120K, alpha_DL_160K, alpha_DL_190K, alpha_DL_220K, alpha_DL_260K, beta_DL_0.10GPa, beta_DL_0.20GPa, beta_DL_0.45GPa, beta_DL_0.56GPa, beta_DL_0.57GPa, beta_DL_0.64GPa, beta_DL_0.67GPa, beta_DL_0.70GPa, beta_DL_0.90GPa, beta_DL_1.28GPa, beta_DL_1.34GPa, beta_DL_1.67GPa, beta_DL_2.10GPa, beta_DL_2.39GPa, beta_DL_2.90GPa. DOI: 10.1107/S2052252523002452/fc5065sup1.cif

Supporting figures and tables. DOI: 10.1107/S2052252523002452/fc5065sup2.pdf

CCDC references: 2206626, 2206627, 2206628, 2206629, 2206630, 2206631, 2206632, 2206633, 2206634, 2206635, 2206636, 2206637, 2206638, 2206639, 2206640, 2206641, 2206642, 2206643, 2206644, 2206645, 2206646, 2206647, 2206648, 2206649, 2206650, 2206651, 2206652, 2206653, 2206654, 2206655, 2206656, 2206657, 2206658

## Figures and Tables

**Table 1:** Selected crystal data of DL-menthol polymorphs α and β determined at 296 K (*cf.* Tables S1 and S2)

Formula		α-DL-C_10_H_20_O	α-DL-C_10_H_20_O	β-DL-C_10_H_20_O	β-DL-C_10_H_20_O
Pressure (GPa)		0.0001	0.60	0.10	0.56
Crystal system		Triclinic	Triclinic	Monoclinic	Monoclinic
Space group		*P* 1	*P* 1	*P*2_1_/*n*	*P*2_1_/*n*
*a* (Å)		12.035 (7)	11.601 (1)	11.928 (6)	11.728 (1)
*b* (Å)		12.536 (6)	12.022 (3)	21.334 (2)	21.085 (1)
*c* (Å)		12.859 (9)	12.596 (2)	12.235 (8)	12.258 (2)
α (°)		117.56 (6)	117.58 (2)	90	90
β (°)		98.61 (5)	99.50 (3)	103.71 (3)	104.60 (5)
γ (°)		103.93 (5)	103.76 (4)	90	90
*V* (Å^3^)		1590.6 (18)	1432.9 (1)	3025 (3)	2933 (3)
*Z*/*Z*′		6/3	6/3	12/3	12/3
*D*_*x*_ (g cm^−3^)		0.979 (1)	1.086 (1)	1.029 (2)	1.062 (2)

## References

[bb1] Adhikari, K., Flurchick, K. M. & Valenzano, L. (2015). *Comput. Theor. Chem.* **1062**, 90–98.

[bb2] Allan, D. R. & Clark, S. J. (1999). *Phys. Rev. B*, **60**, 6328–6334.

[bb3] Allan, D. R., Clark, S. J., Brugmans, M. J. P., Ackland, G. J. & Vos, W. L. (1998). *Phys. Rev. B*, **58**, R11809–R11812.

[bb5] Andrzejewski, M., Casati, N. & Katrusiak, A. (2017). *Dalton Trans.* **46**, 14795–14803. 10.1039/c7dt02511d 29048089

[bb4] Andrzejewski, M. & Katrusiak, A. (2017). *J. Phys. Chem. Lett.* **8**, 929–935. 10.1021/acs.jpclett.7b00019 28170266

[bb6] Anioła, M. & Katrusiak, A. (2015). *Cryst. Growth Des.* **15**, 764–770.

[bb7] Baughman, R. H., Stafström, S., Cui, C. & Dantas, S. O. (1998). *Science*, **279**, 1522–1524. 10.1126/science.279.5356.1522 9488648

[bb8] Bentley, R. (2006). *Chem. Rev.* **106**, 4099–4112. 10.1021/cr050049t 16967929

[bb9] Bhardwaj, R. M., McMahon, J. A., Nyman, J., Price, L. S., Konar, S., Oswald, I. D. H., Pulham, C. R., Price, S. L. & Reutzel-Edens, S. M. (2019). *J. Am. Chem. Soc.* **141**, 13887–13897. 10.1021/jacs.9b06634 31394896

[bb10] Bhatia, S. P., McGinty, D., Letizia, C. S. & Api, A. M. (2008). *Food Chem. Toxicol.* **46**, S215–S217. 10.1016/j.fct.2008.06.039 18640220

[bb11] Boldyreva, E. V., Shakhtshneider, T. P., Ahsbahs, H., Sowa, H. & Uchtmann, H. (2002). *J. Therm. Anal. Calorim.* **68**, 437–452.

[bb12] Bombicz, P., Buschmann, J., Luger, P., Dung, N. X. & Nam, C. B. (1999). *Z. Kristallogr.* **214**, 420–423.

[bb13] Bridgman, P. W. (1949). *The Physics of High Pressure*, pp. 208–210. London: Bell and Sons.

[bb14] Bridgman, P. W. (1964). *Collected Experimental Papers.* Cambridge, MA: Harvard University Press.

[bb103] Brock, C. P. & Duncan, L. L. (1994). *Chem. Mater.* **6**, 1307–1312.

[bb15] Budzianowski, A. & Katrusiak, A. (2004). In *High-Pressure Crystallography*, edited by A. Katrusiak and P. McMillan. NATO Science Series (Series II: Mathematics, Physics and Chemistry), Vol. 140, pp 101–112. Dordrecht: Springer.

[bb16] Bujak, M., Podsiadło, M. & Katrusiak, A. (2008). *J. Phys. Chem. B*, **112**, 1184–1188. 10.1021/jp075491p 18181597

[bb17] Bujak, M., Podsiadło, M. & Katrusiak, A. (2018). *CrystEngComm*, **20**, 328–333.

[bb18] Cai, W., Marciniak, J., Andrzejewski, M. & Katrusiak, A. (2013). *J. Phys. Chem. C*, **117**, 7279–7285.

[bb101] Cliffe, M. J. & Goodwin, A. L. (2012). *J. Appl. Cryst.* **45**, 1321–1329.

[bb19] Coleman, W. M., Perfetti, T. A. & Suber, R. L. (1998). *J. Chromatogr. Sci.* **36**, 318–321.

[bb20] Corvis, Y., Négrier, P., Massip, S., Leger, J. M. & Espeau, P. (2012). *CrystEngComm*, **14**, 7055–7064.

[bb21] Corvis, Y., Wurm, A., Schick, C. & Espeau, P. (2015). *CrystEngComm*, **17**, 5357–5359.

[bb22] Rigaku Oxford Diffraction (2015). *CrysAlisPro.* Rigaku Corporation, The Woodlands, TX, USA.

[bb23] Dolomanov, O. V., Bourhis, L. J., Gildea, R. J., Howard, J. A. K. & Puschmann, H. (2009). *J. Appl. Cryst.* **42**, 339–341.

[bb24] Fedorov, A. Y., Rychkov, D. A., Losev, E. A., Zakharov, B. A., Stare, J. & Boldyreva, E. V. (2017). *CrystEngComm*, **19**, 2243–2252.

[bb25] Feynman, R. P. (1972). *Statistical Machanics. A Set of Lectures.* New York: W. A. Benjamin Inc.

[bb26] Groom, C. R., Bruno, I. J., Lightfoot, M. P. & Ward, S. C. (2016). *Acta Cryst.* B**72**, 171–179. 10.1107/S2052520616003954 PMC4822653 27048719

[bb27] Hazen, R. M. & Finger, L. (1982). *Comparative Crystal Chemistry*. New York: John Wiley.

[bb28] Hopp, R. & Lawrence, B. M. (2006). *Mint: the Genus Mentha*, edited by B. Lawrence, pp. 371–399. Boca Raton: CRC Press.

[bb29] Jacques, J., Collet, A. & Wilen, S. H. (1981). *Enantiomers, Racemates and Resolution.* New York: Wiley.

[bb30] Katrusiak, A. (2008). *Acta Cryst.* A**64**, 135–148. 10.1107/S0108767307061181 18156679

[bb31] Katrusiak, A., Szafrański, M. & Podsiadło, M. (2011). *Chem. Commun.* **47**, 2107–2109. 10.1039/c0cc02630a 21183983

[bb32] Kaźmierczak, M. & Katrusiak, A. (2013). *J. Phys. Chem. C*, **117**, 1441–1446.

[bb33] Kitaigorodsky, A. I. (1973). *Molecular Crystals and Molecules.* New York, London: Academic Press.

[bb34] Kofoed, P. M., Hoser, A. A., Diness, F., Capelli, S. C. & Madsen, A. Ø. (2019). *IUCrJ*, **6**, 558–571. 10.1107/S2052252519003014 PMC6608639 31316801

[bb35] Krawczyk, S. & Gdaniec, M. (2005). *Acta Cryst.* E**61**, o4116–o4118.

[bb36] Kuhnert-Brandstätter, M., Ulmer, R. & Langhammer, L. (1974). *Arch. Pharm. Pharm. Med. Chem.* **307**, 497–503. 10.1002/ardp.19743070702 4849989

[bb102] Langreiter, T. & Kahlenberg, V. (2015). *Crystals*, **5**, 143–153.

[bb37] Lee, Y., Vogt, T., Hriljac, J. A., Parise, J. B. & Artioli, G. (2002). *J. Am. Chem. Soc.* **124**, 5466–5475. 10.1021/ja0255960 11996588

[bb38] Li, Q., Li, S., Wang, K., Liu, J., Yang, K., Liu, B., Zou, G. & Zou, B. (2014). *J. Phys. Chem. C*, **118**, 5848–5853.

[bb39] Macrae, C. F., Sovago, I., Cottrell, S. J., Galek, P. T. A., McCabe, P., Pidcock, E., Platings, M., Shields, G. P., Stevens, J. S., Towler, M. & Wood, P. A. (2020). *J. Appl. Cryst.* **53**, 226–235. 10.1107/S1600576719014092 PMC6998782 32047413

[bb40] Madsen, A. Ø. & Larsen, S. (2007). *Angew. Chem. Int. Ed.* **46**, 8609–8613. 10.1002/anie.200702423 17918271

[bb41] Mao, H. K., Xu, J. & Bell, P. M. (1986). *J. Geophys. Res.* **91**, 4673–4676.

[bb42] Marciniak, J., Andrzejewski, M., Cai, W. & Katrusiak, A. (2014). *J. Phys. Chem. C*, **118**, 4309–4313.

[bb43] McKemy, D. D., Neuhausser, W. M. & Julius, D. (2002). *Nature*, **416**, 52–58. 10.1038/nature719 11882888

[bb44] Merrill, L. & Bassett, W. A. (1974). *Rev. Sci. Instrum.* **45**, 290–294.

[bb45] Nelyubina, Y. V., Glukhov, I. V., Antipin, M. Y. & Lyssenko, K. A. (2010). *Chem. Commun.* **46**, 3469–3471. 10.1039/b927429d 20407690

[bb46] Newnham, R. E. (2005). *Properties of Materials: Anisotropy, Symmetry, Structure*, p. 7. Oxford University Press.

[bb47] Nicolaou, Z. & Motter, A. (2012). *Nat. Mater.* **11**, 608–613. 10.1038/nmat3331 22609557

[bb48] Nye, J. F. (1984). *Physical Properties of Crystals: Their Representation by Tensors and Matrices*, pp. 145–146. Oxford: Clarendon Press.

[bb49] Olejniczak, A., Katrusiak, A., Podsiadło, M. & Katrusiak, A. (2022*a*). *IUCrJ*, **9**, 49–54. 10.1107/S2052252521010381 PMC8733875 35059209

[bb50] Olejniczak, A., Katrusiak, A., Podsiadło, M. & Katrusiak, A. (2022*b*). *Cryst. Growth Des.* **22**, 5996–6003.

[bb51] O’Neil, M. J. (2013). *The Merck Index – an Encyclopedia of Chemicals, Drugs, and Biologicals* Cambridge: Royal Society of Chemistry.

[bb52] Paliwoda, D., Dziubek, K. F. & Katrusiak, A. (2012). *Cryst. Growth Des.* **12**, 4302–4305.

[bb53] Piermarini, G. J., Block, S., Barnett, J. D. & Forman, R. A. (1975). *J. Appl. Phys.* **46**, 2774–2780.

[bb54] Price, L. S. & Price, S. L. (2022). *Cryst. Growth Des.* **22**, 1801–1816. 10.1021/acs.cgd.1c01381 PMC9097456 35571354

[bb55] Ramsay, I. W. & Rogers, D. (1952). *Acta Cryst.* **5**, 268–271.

[bb56] Reichl, L. E. (1998). *A Modern Course in Statistical Physics.* New York: John Wiley.

[bb57] Roszak, K. & Katrusiak, A. (2021). *Acta Cryst.* B**77**, 449–455. 10.1107/S205252062100398X 34096527

[bb58] Roszak, K., Katrusiak, A. & Katrusiak, A. (2016). *Cryst. Growth Des.* **16**, 3947–3953.

[bb59] Safari, F. & Katrusiak, A. (2021). *J. Phys. Chem. C*, **125**, 23501–23509. 10.1021/acs.jpcc.1c07297 PMC8559611 34737842

[bb60] Sell, C. (1999). *The Chemistry of Fragrances: From Perfumer to Consumer*, pp. 51–124. Cambridge: The Royal Society of Chemistry.

[bb61] Sheldrick, G. M. (2015*a*). *Acta Cryst.* C**71**, 3–8.

[bb62] Sheldrick, G. M. (2015*b*). *Acta Cryst.* A**71**, 3–8.

[bb63] Skumiel, J. (2010). MSc thesis. Adam Mickiewicz University, Poland.

[bb64] Sobczak, S. & Katrusiak, A. (2017). *J. Phys. Chem. C*, **121**, 2539–2545.

[bb65] Sobczak, S., Ratajczyk, P. & Katrusiak, A. (2021). *Chem. Eur. J.* **27**, 10769–10779.

[bb66] Tchoń, D., Bowskill, D., Sugden, I., Piotrowski, P. & Makal, A. (2021). *J. Mater. Chem. C.* **9**, 2491–2503.

[bb67] Weineerg, B. (1908). *Nature*, **78**, 390.

[bb68] Wright, F. E. (1917). *J. Am. Chem. Soc.* **39**, 1515–1524.

[bb69] Yalkowsky, S. H., He, Y. & Jain, P. (2010). *Handbook of Aqueous Solubility Data.* Boca Raton: CRC Press.

[bb70] Zhang, Y., Yao, M., Du, M., Yao, Z., Wang, Y., Dong, J., Yang, Z., Sundqvist, B., Kováts, É., Pekker, S. & Liu, B. (2020). *J. Am. Chem. Soc.* **142**, 7584–7590. 10.1021/jacs.0c01703 32250116

[bb71] Zieliński, W. & Katrusiak, A. (2013). *Cryst. Growth Des.* **13**, 696–700.

[bb72] Zieliński, W. & Katrusiak, A. (2015). *CrystEngComm*, **17**, 5468–5473.

