# Supplementary material for: Low-density preference of the ambient and high-pressure polymorphs of dl-menthol
Source: IUCrJ. 2023 Apr 21;10(Pt 3):341–51. doi: 10.1107/S2052252523002452 (PMC10161766; doi:10.1107/S2052252523002452)
Supplement: Supplementary file 2 [file m-10-00341-sup2.pdf]

IUCrJ

Volume 10 (2023)

Supporting information for article:

Low-density preference of the ambient and high-pressure polymorphs of dl-menthol

Kinga Roszak and Andrzej Katrusiak

S1. Tables

S1.1. Crystallographic details

Table S1 Selected crystallographic data of α-*DL*-Menthol

Pressure (GPa)		0.001	0.001	0.001	0.001	0.001
Temperature (K)		120	160	190	220	260
Formula weight		156.26	156.26	156.26	156.26	156.26
Wavelength (Å)		0.71073	0.71073	0.71073	0.71073	0.71073
Crystal system		Triclinic	Triclinic	Triclinic	Triclinic	Triclinic
Space group		$P\bar{1}$	$P\bar{1}$	$P\bar{1}$	$P\bar{1}$	$P\bar{1}$
Unit cell dimensions (Å,°)	*a*	12.0054(11)	11.9503(12)	11.9443(11)	11.9423(11)	11.9801(14)
	b	12.2439(10)	12.3527(10)	12.4081(9)	12.4458(11)	12.5078(12)
	c	12.6778(9)	12.7242(9)	12.7491(10)	12.7755(11)	12.8269(16)
	α	117.176(7)	117.256(7)	117.321(8)	117.352(9)	117.531(11)
	β	100.286(7)	99.818(7)	99.504(7)	99.261(7)	98.822(10)
	γ	103.377(7)	103.584(8)	103.673(7)	103.787(8)	103.945(9)
Volume ($Å^3$)		1523.4(2)	1536.7(2)	1547.0(2)	1555.4(3)	1573.9(4)
Z		6	6	6	6	6
Calculated density (gcm^{-3})		1.022	1.013	1.006	1.001	0.989
Absorption coefficient (mm^{-1})		0.063	0.062	0.062	0.062	0.061
F(000)		528	528	528	528	528
Crystal size (mm)		0.25·0.10·0.05	0.25·0.10·0.05	0.25·0.10·0.05	0.25·0.10·0.05	0.25·0.10·0.05
Θ-range for data collection (°)		1.85 to 26.24	1.85 to 26.26	1.85 to 26.24	1.85 to 26.26	1.84 to 26.27
Min/max indices: h, k, l		-14/13, -15/15, -11/15	-14/13, -15/15, -11/15	-14/13, -15/15, -11/15	-14/13, -15/15, -11/15	-14/13, -15/15, -11/15
Reflect. Collected/unique		11319/5915	11620/5994	11808/6036	11870/6062	11914/6121
R_{int}/ Completeness (%)		0.0443/96.3	0.0451/96.5	0.0351/96.7	0.0308/96.4	0.0615/96.2
Data/restrains/parameters		5915/206/335	5994/16/318	6036/14/314	6062/14/318	6124/14/314
Goodness-of-fit on F^2		1.091	1.057	1.050	1.044	0.927
Final R_1/wR_2 (I>2δ$_1$)		0.0609/0.1714	0.0615/0.1635	0.0620/0.1630	0.0613/0.1628	0.0826/0.2164
R_1/wR^2 (all data)		0.1079/0.2202	0.1098/0.2119	0.1098/0.2037	0.1165/0.2059	0.2232/0.3464

Table S1 continued. Selected crystallographic data of α-*DL*-Menthol

Pressure (GPa)		0.001	0.10	0.15	0.30	0.51
Temperature (K)		296	296	296	296	296
Formula weight		156.26	156.26	156.26	156.26	156.26
Wavelength (Å)		0.71073	0.71073	0.71073	0.71073	0.71073
Crystal system		Triclinic	Triclinic	Triclinic	Triclinic	Triclinic
Space group		$P\bar{1}$	$P\bar{1}$	$P\bar{1}$	$P\bar{1}$	$P\bar{1}$
Unit cell dimensions (Å,°)	*a*	12.035(7)	11.933(2)	11.923(6)	11.886(2)	11.735(8)
	b	12.536(6)	12.489(3)	12.429(2)	12.233(3)	12.0842(15)
	c	12.859(9)	12.767(3)	12.770(2)	12.647(3)	12.571(2)
	α	117.56(6)	117.43(3)	117.48(2)	117.45(3)	117.42(1)

	β	98.61(5)	98.77(2)	99.16(3)	99.624(18)	99.67(10)
	γ	103.93(5)	104.13(2)	103.81(3)	103.81(2)	103.75(9)
Volume (Å^3)		1590.6(18)	1558.3(7)	1548.7(9)	1500.5(8)	1455.3(18)
Z		6	6	6	6	6
Calculated density (gcm^{-3})		0.979	0.999	1.005	1.035	1.083
Absorption coefficient (mm^{-1})		0.060	0.062	0.062	0.064	0.066
F(000)		528	528	528	528	528
Crystal size (mm)		0.23·0.20·0.08	0.40·0.30·0.05	0.45·0.45·0.05	0.40·0.30·0.05	0.38·0.33·0.05
Θ-range for data collection (°)		1.79 to 24.999	5.78 to 26.28	4.803 to 26.21	4.84 to 26.67	3.102 to 28.38
Min/max indices: h, k, l		-14/13,-14/14, -11/15	-14/14, -6/6, -12/12	-6/6, -15/15, -15/15	-14/14, -6/6, -12/12	-13/13,-6/6, -14/14
Reflect. Collected/unique		11264/5556	7351/1392	7285/1388	7233/1384	8522/1421
R_{int}/Completeness (%)		0.0322/99.2	0.0833/22.0	0.0852/22.3	0.0791/21.7	0.3725/19.5
Data/ restrains/parameters		5556/206/314	1392/207/314	1388/208/314	1378/207/314	1421/207/314
Goodness-of-fit on F^2		0.999	1.024	1.037	1.015	0.951
Final R_1/wR_2 (I>2δ$_1$)		0.0585/0.1559	0.0715/0.1735	0.0789/0.2064	0.0669/0.1696	0.0697/0.1194
R_1/ wR_2 (all data)		0.1084/0.1851	0.1621/0.2202	0.1661/0.2676	0.1578/0.2276	0.3417/0.2094

Table S1 continued. Selected crystallographic data of α-*DL*-Menthol

Pressure (GPa)		0.60	0.65	0.84	1.12	1.44
Temperature (K)		296	296	296	296	296
Formula weight		156.26	156.26	156.26	156.26	156.26
Wavelength (Å)		0.71073	0.71073	0.71073	0.71073	0.71073
Crystal system		Triclinic	Triclinic	Triclinic	Triclinic	Triclinic
Space group		$P\bar{1}$	$P\bar{1}$	$P\bar{1}$	$P\bar{1}$	$P\bar{1}$
Unit cell dimensions (Å,°)	*a*	11.601(10)	11.677(6)	11.603(6)	11.441(3)	11.340(2)
	b	12.022(3)	11.9875(14)	11.8897(10)	11.726(4)	11.612(1)
	c	12.596(2)	12.442(9)	12.368(9)	12.238(4)	12.094(3)
	α	117.58(2)	117.25(3)	117.791(11)	117.51(3)	117.75(1)
	β	99.50(3)	99.96(2)	99.44(5)	99.78(2)	99.55(2)

	γ	103.76(4)	103.324(12)	103.16(3)	102.45(3)	101.95(3)
Volume (Å^3)		1432.9(13)	1426.4(14)	1395.7(13)	1351.4(9)	1314.0(5)
Z		6	6	6	6	6
Calculated density (gcm^{-3})		1.086	1.091	1.115	1.150	1.185
Absorption coefficient (mm^{-1})		0.067	0.067	0.069	0.071	0.073
F(000)		528	528	528	528	528
Crystal size (mm)		0.45·0.45·0.05	0.39·0.30·0.06	0.40·0.30·0.06	0.40·0.30·0.06	0.39·0.30·0.06
Θ-range for data collection (°)		4.95 to 26.66	3.118 to 28.42	3.128 to 28.73	3.144 to 29.05	3.162 to 29.506
Min/max indices: h, k, l		-6/6, -15/15, -15/15	-13/13,-6/6, -14/14	-13/13,-6/6, -14/14	-13/13,-6/6, -14/14	-13/13,-6/6, -14/14
Reflect. Collected/unique		6663/1268	8431/1405	8446/1407	8413/1404	8447/1409
R_{int}/Completeness (%)		0.1006/21.0	0.3894/19.5	0.3747/19.5	0.3835/19.4	0.3775/19.3
Data/restrains/parameters		1268/207/314	1405/246/318	1407/246/318	1404/246/318	1409/243/317
Goodness-of-fit on F^2		1.015	0.945	0.965	0.944	0.941
Final R_1/wR_2 (I>2δ$_1$)		0.0643/0.1566	0.0718/0.1228	0.0928/0.2018	0.0662/0.1088	0.0722/0.1477
R_1/wR_2 (all data)		0.1531/0.2033	0.3507/0.2147	0.3497/0.3254	0.3486/0.1955	0.3502/0.2643

Table S1 continued. Selected crystallographic data of α-*DL*-Menthol

Pressure (GPa)		1.70	2.10	3.37
Temperature (K)		296	296	296
Formula weight		156.26	156.26	156.26
Wavelength (Å)		0.71073	0.71073	0.71073
Crystal system		Triclinic	Triclinic	Triclinic
Space group		$P\bar{1}$	$P\bar{1}$	$P\bar{1}$
Unit cell dimensions (Å,°)	a	11.262(3)	11.198(3)	11.166(4)
	b	11.533(4)	11.459(4)	11.346(4)
	c	11.991(4)	11.898(4)	11.760(6)
	α	117.74(3)	117.74(4)	118.10(4)
	β	99.47(2)	99.72(2)	99.90(5)
	γ	101.75(3)	101.39(3)	100.68(4)

Volume (Å^3)	1287.7(8)	1263.5(8)	1232.3(9)
Z	6	6	6
Calculated density (gcm^{-3})	1.209	1.232	1.263
Absorption coefficient (mm^{-1})	0.075	0.076	0.078
F(000)	528	528	528
Crystal size (mm)	0.39·0.30·0.06	0.39·0.30·0.06	0.30·0.30·0.06
Θ-range for data collection (°)	3.182 to 29.775	3.196 to 30.038	3.228 to 30.258
Min/max indices: h, k, l	-13/13,-6/6, -14/14	-13/13,-6/6, -14/14	-13/13,-6/6, -14/14
Reflect. Collected/unique	8430/1408	8522/1420	8331/1391
R_{int}/Completeness (%)	0.3725/19.1	0.3837/19.3	0.3885/18.8
Data/restrains/parameters	1408/243/317	1420/246/318	1391/245/317
Goodness-of-fit on F^2	0.944	0.957	0.955
Final R_1/wR_2 (I>2δ$_1$)	0.0720/0.1376	0.0720/0.1376	0.0935/0.2124
R_1/wR_2 (all data)	0.3512/0.2496	0.3517/0.2496	0.3643/0.3565

Table S2 Selected crystallographic data of β-*DL*-menthol

Pressure (GPa)		0.10	0.20	0.45	0.56	0.57
Temperature (K)		296	296	296	296	296
Formula weight		156.26	156.26	156.26	156.26	156.26
Wavelength (Å)		0.71073	0.71073	0.71073	0.71073	0.71073
Crystal system		Monoclinic	Monoclinic	Monoclinic	Monoclinic	Monoclinic
Space group		$P2_1/n$	$P2_1/n$	$P2_1/n$	$P2_1/n$	$P2_1/n$
Unit cell dimensions (Å,°)	a	11.928(6)	12.031(4)	11.855(4)	11.728(11)	11.707(10)
	b	21.3338(19)	21.333(1)	21.114(1)	21.085(1)	20.974(3)
	c	12.235(8)	12.212(5)	12.228(4)	12.258(2)	11.987(10)
	$α$	90	90	90	90	90
	$β$	103.71(3)	104.03(5)	104.37(4)	104.60(2)	103.48(10)
	$γ$	90	90	90	90	90
Volume (Å^3)		3025(3)	3040.9(18)	2964.9(15)	2933(3)	2862(4)
Z		12	12	12	12	12
Calculated density (gcm^{-3})		1.029	1.024	1.050	1.062	1.088
Absorption coefficient (mm^{-1})		0.063	0.063	0.065	0.065	0.067

F(000)	1056	1056	1056	1056	1056
Crystal size (mm)	0.45·0.25·0.02	0.45·0.35·0.02	0.48·0.48·0.05	0.40·0.40·0.05	0.45·0.45·0.02
Θ-range for data collection (°)	4.62 to 26.778	4.63 to 26.659	4.23 to 27.095	2.89 to 28.35	3.36 to 26.62
Min/max indices: h, k, l	-10/10, -26/26, -9/9	-10/10, -26/26, -9/9	-10/10, -26/27, -10/10	-5/5, -28/27, -16/16	-10/10, -27/27, -10/10
Reflect. Collected/unique	14374/1506	14702/1533	14984/1684	16901/1645	16614/1419
R_{int}/Completeness (%)	0.1675/23.3	0.1566/24.0	0.0671/25.7	0.0823/22.3	0.1888/19.8
Data/restrains/parameters	1506/189/296	1533/206/312	1684/8/311	1645/205/312	1419/206/312
Goodness-of-fit on F^2	1.006	1.017	1.033	1.013	1.074
Final R_1/wR_2 (I>2δ$_1$)	0.0698/0.1513	0.0733/0.1895	0.0641/0.1609	0.0661/0.1709	0.0946/0.2444
R_1/wR^2 (all data)	0.2039/0.2196	0.2074/0.2826	0.1315/0.1994	0.1668/0.2324	0.2126/0.3475

Table S2 continued Selected crystallographic data of β-*DL*-Menthol

Pressure (GPa)		0.64	0.67	0.70	0.90	1.28
Temperature (K)		296	296	296	296	296
Formula weight		156.26	156.26	156.26	156.26	156.26
Wavelength (Å)		0.71073	0.71073	0.71073	0.71073	0.71073
Crystal system		Monoclinic	Monoclinic	Monoclinic	Monoclinic	Monoclinic
Space group		$P2_1/n$	$P2_1/n$	$P2_1/n$	$P2_1/n$	$P2_1/n$
Unit cell dimensions (Å,°)	*a*	11.638(5)	11.745(5)	11.7042(12)	11.665(4)	11.528(2)
	b	21.198(6)	21.172(3)	21.036(3)	20.8990(9)	20.785(6)
	c	12.2482(19)	12.054(8)	12.178(3)	12.025(4)	11.959(5)
	α	90	90	90	90	90
	β	104.36(2)	104.29(3)	104.505(14)	104.25(4)	104.36(3)
	γ	90	90	90	90	90
Volume (Å^3)		2927.4(16)	2905(2)	2902.8(8)	2841.2(14)	2775.9(15)
Z		12	12	12	12	12
Calculated density (gcm^{-3})		1.064	1.072	1.073	1.096	1.122
Absorption coefficient (mm^{-1})		0.066	0.066	0.066	0.068	0.069
F(000)		1056	1056	1056	1056	1056
Crystal size (mm)		0.45·0.45·0.05	0.45·0.45·0.02	0.45·0.45·0.05	0.45·0.45·0.02	0.45·0.45·0.05
Θ-range for data collection (°)		5.01 to 26.35	3.37 to 22.00	4.78 to 26.38	4.28 to 27.11	4.84 to 26.43

Min/max indices: h, k, l	-10/10, -21/21, -15/14	-10/10, -21/22, -9/9	-14/14, -21/21, -10/10	-9/9, -26/26, -10/10	-13/14, -20/21, -10/10
Reflect. Collected/unique	13201/2311	20654/1202	13598/2354	14619/1440	12745/2237
R_{int}/Completeness (%)	0.1339/38.6	0.2763/33.7	0.0895/39.5	0.1101/23.0	0.1346/39.3
Data/restrains/parameters	2311/207/312	1202/207/313	2354/206/312	1440/206/312	2237/207/312
Goodness-of-fit on F^2	0.900	1.039	1.056	1.040	0.994
Final R_1/wR_2 (I>2δ$_1$)	0.0832/0.2043	0.0994/0.2207	0.0787/0.2127	0.0851/0.2196	0.0918/0.2398
R_1/wR^2 (all data)	0.2379/0.3011	0.2086/0.3070	0.1718/0.2725	0.1667/0.2875	0.2046/0.3223

Table S2 continued Selected crystallographic data of β-*DL*-Menthol

Pressure (GPa)		1.34	1.67	2.10	2.39	2.90
Temperature (K)		296	296	296	296	296
Formula weight		156.26	156.26	156.26	156.26	156.26
Wavelength (Å)		0.71073	0.71073	0.71073	0.71073	0.71073
Crystal system		Monoclinic	Monoclinic	Monoclinic	Monoclinic	Monoclinic
Space group		$P2_1/n$	$P2_1/n$	$P2_1/n$	$P2_1/n$	$P2_1/n$
Unit cell dimensions (Å,°)	*a*	11.529(6)	11.48(2)	11.24(2)	11.382(2)	11.171(3)
	b	20.7315(15)	20.557(6)	20.451(5)	20.381(5)	20.436(8)
	c	11.880(6)	11.79(2)	11.661(18)	11.656(3)	11.620(4)
	α	90	90	90	90	90
	β	104.42(2)	103.2(2)	103.31(7)	103.21(2)	102.80(3)
	γ	90	90	90	90	90
Volume (Å^3)		2750(2)	2709(11)	2609(6)	2632.4(10)	2586.7(15)
Z		12	12	12	12	12
Calculated density (gcm^{-3})		1.132	1.149	1.194	1.183	1.204
Absorption coefficient (mm^{-1})		0.070	0.071	0.074	0.073	0.074
F(000)		1056	1056	1056	1056	1056
Crystal size (mm)		0.45·0.45·0.02	0.45·0.45·0.02	0.45·0.45·0.02	0.45·0.45·0.05	0.45·0.45·0.02
Θ-range for data collection (°)		4.31 to 27.27	4.35 to 26.78	4.37 to 27.13	5.61 to 25.72	4.84 to 26.65
Min/max indices: *h*, *k*, *l*		-9/9, -26/26, -10/10	-9/9, -26/26, -10/10	-9/9, -26/25, -10/10	-13/13, -20/20, -10/10	-9/9, -25/25, -10/10
Reflect. Collected/unique		14113/1401	12550/1267	12410/1252	10113/1917	8700/1145

R_{int}/Completeness (%)	0.1157/22.7	0.1894/21.9	0.2412/21.7	0.1643/38.2	0.2272/21.0
Data/restrains/parameters	1401/206/312	1267/206/311	1252/206/312	1917/208/311	1145/208/312
Goodness-of-fit on F^2	1.062	0.976	1.020	0.939	1.038
Final R_1/wR_2 (I>2δ$_1$)	0.0976/0.2542	0.0806/0.1921	0.0945/0.2348	0.0916/0.2188	0.0989/0.1908
R_1/wR^2 (all data)	0.1776/0.3299	0.2086/0.2936	0.2255/0.3406	0.2277/0.3173	0.2638/0.2846

Table S3 Average coefficients of compressibility for α-*DL*-menthol, related to crystallographic axes, calculated in the all range between 0.1 MPa and 2.1 GPa along with Birch-Murnaghan coefficients (Cliffe & Goodwin, 2012).

axes	K(TPa^{-1})	σK(TPa^{-1})	direction			Empirical parameters			
			a	*b*	*c*	*ε0*	*λ*	*Pc*	*v*
**X1**	45.1622	1.7299	0.5902	-0.6293	0.5056	5.6348e+00	-5.6615e+00	-0.6869	0.0121
**X2**	33.2731	1.9106	-0.4299	-0.6918	-0.5801	1.2166e+00	-1.2558e+00	-0.3394	0.0311
**X3**	26.7064	3.4626	-0.7955	-0.0388	0.6047	1.1540e+01	-1.1541e+01	-0.9070	0.0040
V	104.7997	8.7586							

Birch-Murnaghan Coefficients

	B$_0$ (GPa)	σB$_0$ (GPa)	V$_0$ (Å^3)	σV$_0$ (Å^3)	B'	σB'	P$_c$ (GPa)
**2nd**	5.7041	0.2530	1575.0648	7.9444	4	n/a	0
**3rd**	3.4673	0.5491	1602.1224	11.3372	8.1782	1.3771	0

Table S4 Average coefficients of compressibility for β-*DL*-menthol, related to crystallographic axes, calculated in the all range between 0.1 MPa and 2.39 GPa along with Birch-Murnaghan coefficients (Cliffe & Goodwin, 2012).

axes	K(TPa^{-1})	σK(TPa^{-1})	direction			Empirical parameters			
			a	*b*	*c*	*ε0*	*λ*	*Pc*	*v*
**X1**	25.2690	3.8137	0.8977	-0.0000	0.4406	1.8286e-01	-2.0825e-01	-0.2591	0.1378
**X2**	21.6087	3.2234	0.0000	-1.0000	0.0000	5.8585e-01	-4.2652e-01	-4.7295	0.2014
**X3**	22.0944	5.8233	0.2965	-0.0000	-0.9550	1.1498e+00	-6.7906e-01	-10.6616	0.2197
**V**	66.4824	4.7848							

Birch-Murnaghan Coefficients

	B_0 (GPa)	σB_0 (GPa)	V_0 ($Å^3$)	σV_0 ($Å^3$)	B'	σB'	P_c (GPa)
**2nd**	9.9084	0.7347	3078.5697	19.3519	4	n/a	0
**3rd**	8.7195	3.2308	3091.5447	43.7668	5.2299	3.4940	0

S2. Figures

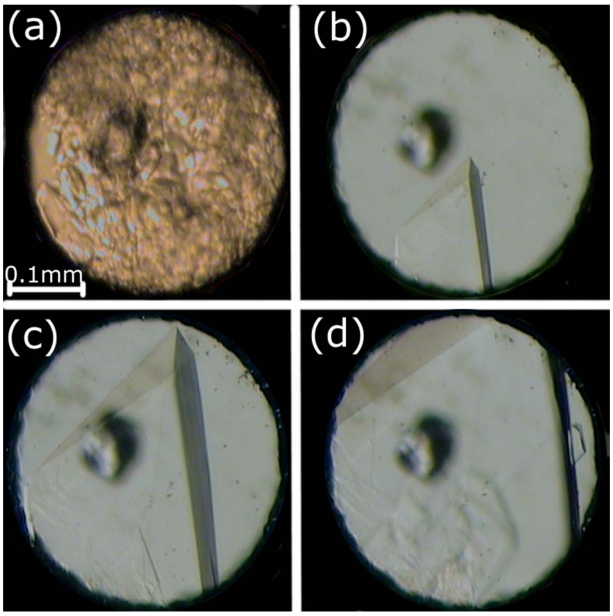

Figure S1 Isochoric growth of the α-*DL*-$C_{10}H_{20}O$ single crystal from methanol:water mixture (1:2 in vol.) at (a) 403 K, (b) 373 K, (c) 313 K (d) 296 K/0.15 GPa. The ruby chip for pressure calibration is located in the middle on the hole.

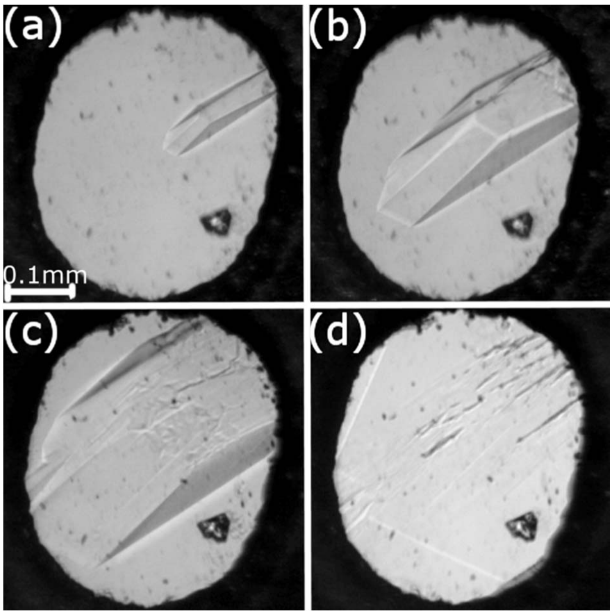

Figure S2 Isochoric growth of the α-*DL*-$C_{10}H_{20}O$ single crystal from methanol:water mixture (1:2 in vol.) at (a) 423 K, (b) 383 K, (c) 353 K (d) 296 K/0.30 GPa. The ruby chip for pressure calibration lies close to the right down corner of DAC chamber.

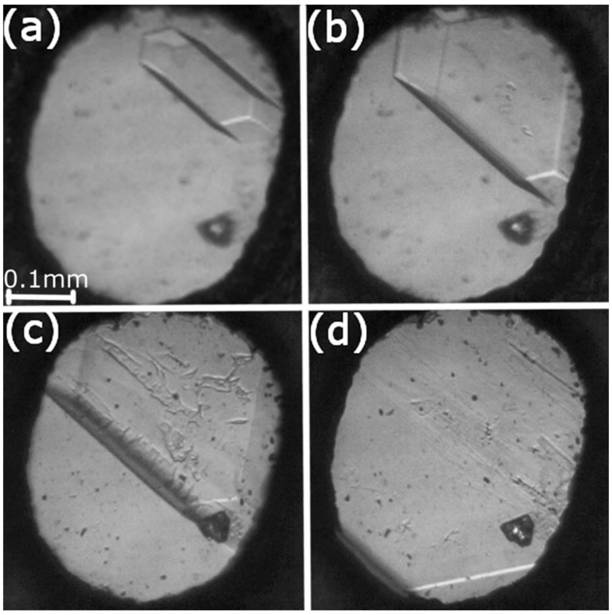

Figure S3 Isochoric growth of the α-*DL*-$C_{10}H_{20}O$ single crystal from methanol:water mixture (1:2 in vol.) at (a) 393 K, (b) 343 K, (c) 303 K, (d) 296 K/0.35 GPa. The ruby chip for pressure calibration lies close to the right down corner of DAC chamber.

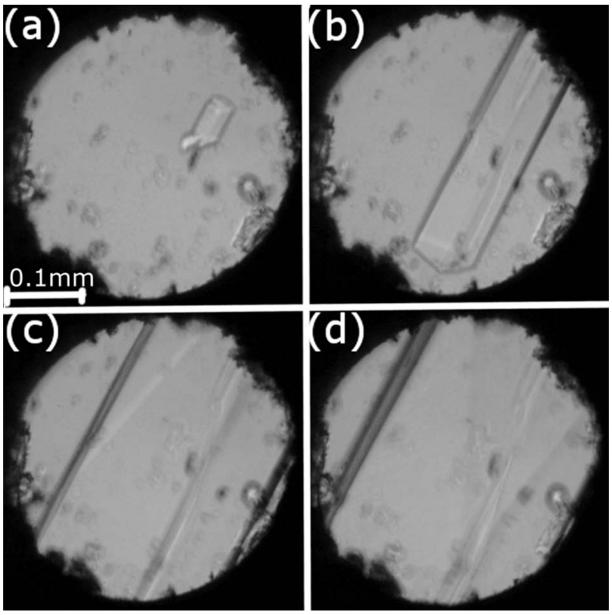

Figure S4 Isochoric growth of the α-*DL*-$C_{10}H_{20}O$ single crystal from methanol:water mixture (1:2 in vol.) at (a) 413 K, (b) 353 K, (c) 323 K, (d) 296 K/0.39 GPa. The ruby chip for pressure calibration lies close to the right down corner of DAC chamber.

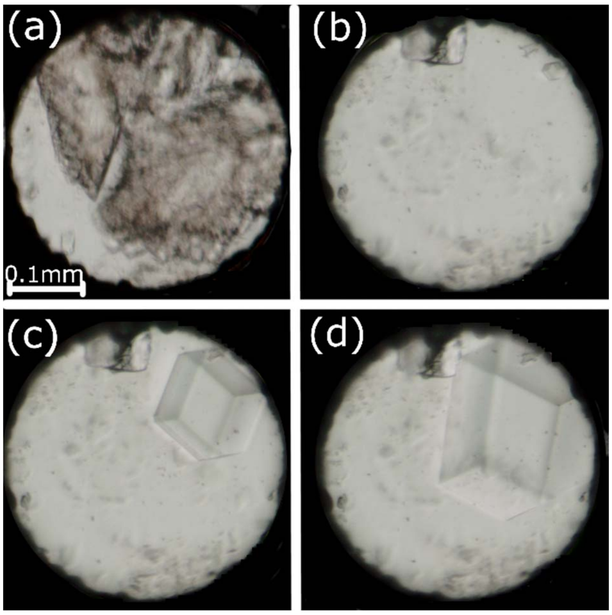

Figure S5 Isochoric growth of the β-*DL*-$C_{10}H_{20}O$ single crystal from methanol:water mixture (1:2 in vol.) at (a) 413 K, (b) 393 K, (c) 343 K, (d) 296 K/0.53 GPa. The ruby chip for pressure calibration lies close to the top corner of DAC chamber.

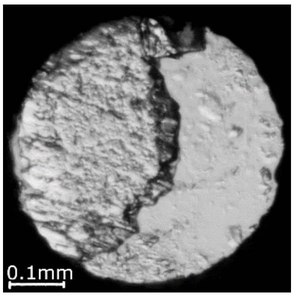

Figure S6 The half-molten β-*DL*-menthol (in the left part of the DAC chamber) at 0.10 GPa/296 K. The crystal was grown from the melt at 0.53 GPa, by lowering the temperature from 393 K.

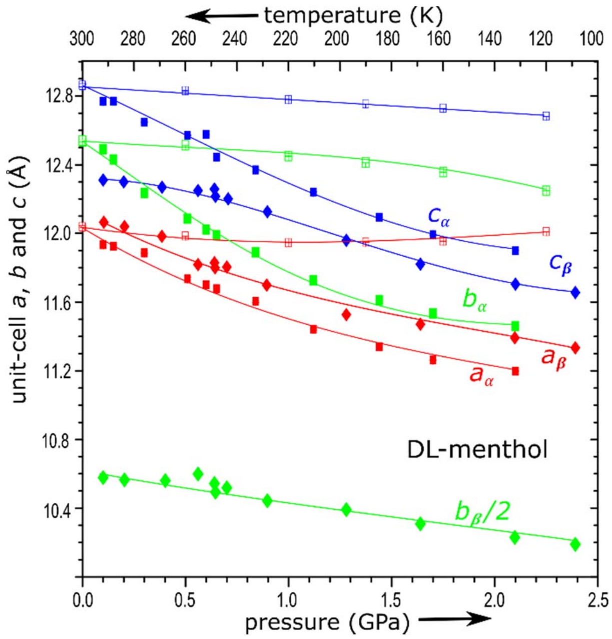

Figure S7 Pressure (full symbols) and temperature (open symbols) dependence of unit-cell parameters *a*, *b*, *c* in α-*DL*-menthol (squares) and β-*DL*-menthol (diamonds) in their original settings (triclinic and monoclinic, respectively). Parameter b_β have been halved for conveniently including it in one plot.

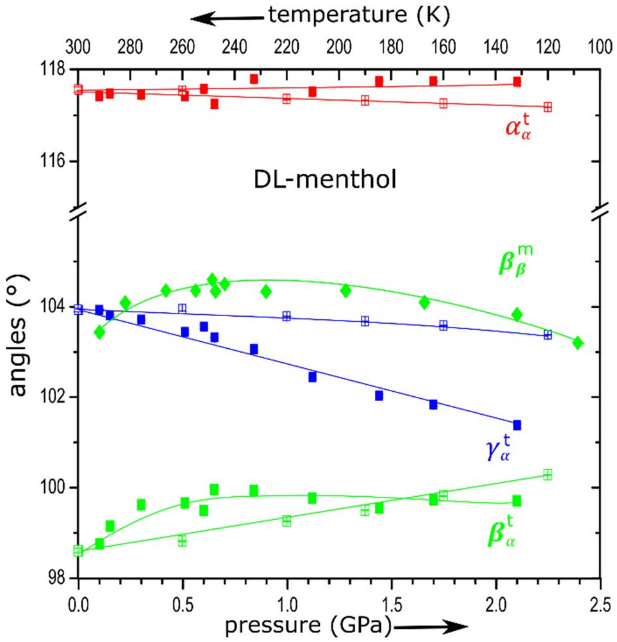

Figure S8 Pressure (full symbols) and temperature (open symbols) dependence of unit-cell angles: α-*DL*-menthol (squares) and β-*DL*-menthol (diamonds). The subscripts mark the triclinic setting for polymorph α and the monoclinic setting of polymorph β.

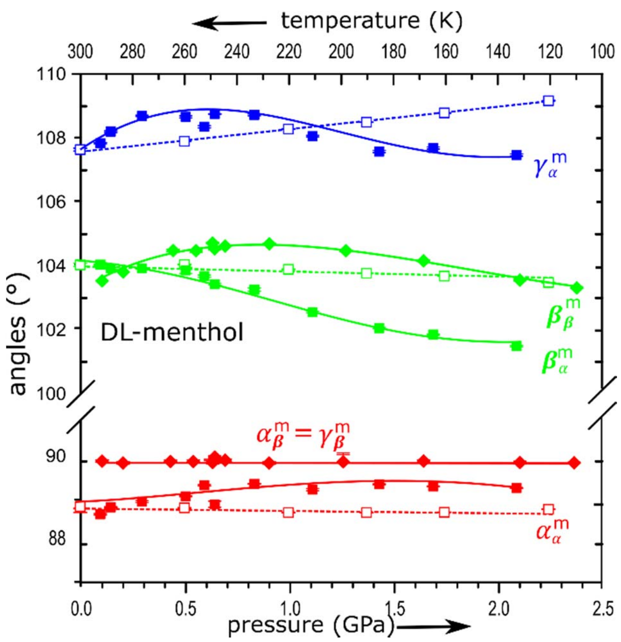

Figure S9 Pressure (full symbols) and temperature (open symbols) dependence of unit-cell angles of α-*DL*-menthol (squares) and β-*DL*-menthol (diamonds, *cf.* Figure S8). For the triclinic polymorph α, the angles of pseudo-monoclinic lattice (Equation 1) are plotted.

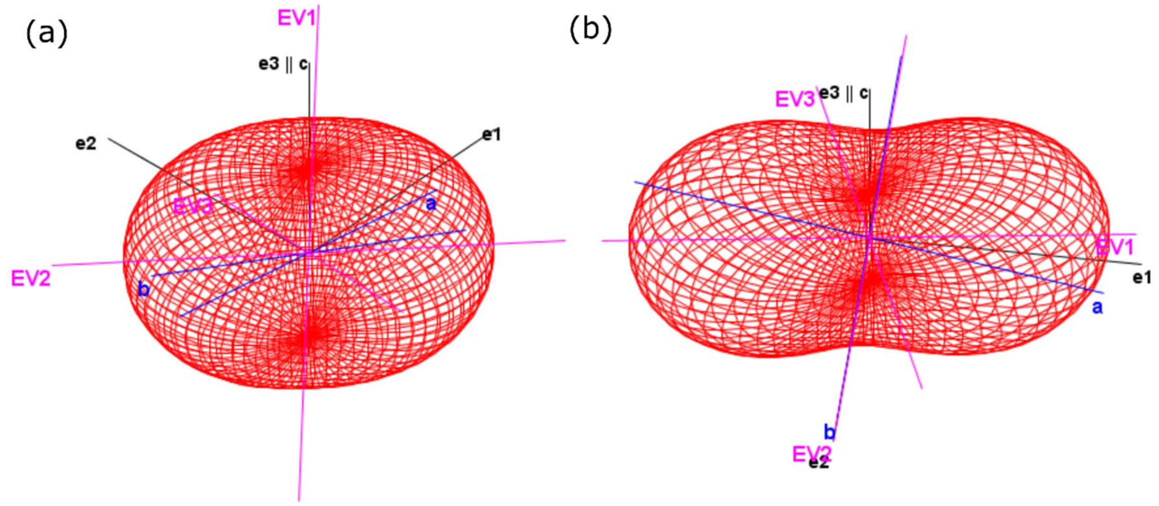

Figure S10 Graphical representations of the compressibilities of *DL*-menthol polymorphs α (a) and β (b), both at 0.4 GPa, drawn by program TEV (Langreiter & Kahlenberg, 2015).

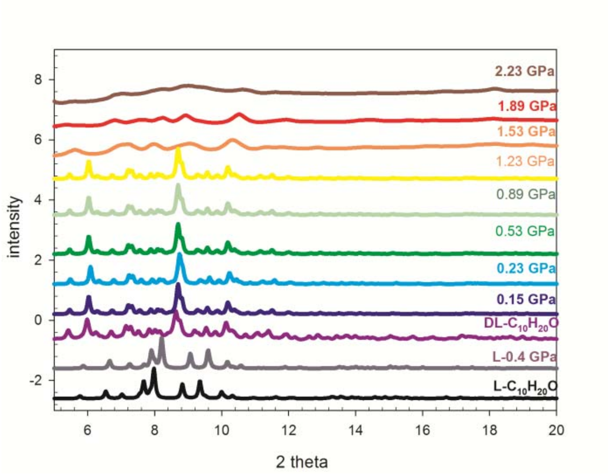

Figure S11 Powder X-ray diffraction patterns generated for *DL*-menthol and those measured for the sample recrystallized *in situ* in the DAC up to 2.23 GPa.

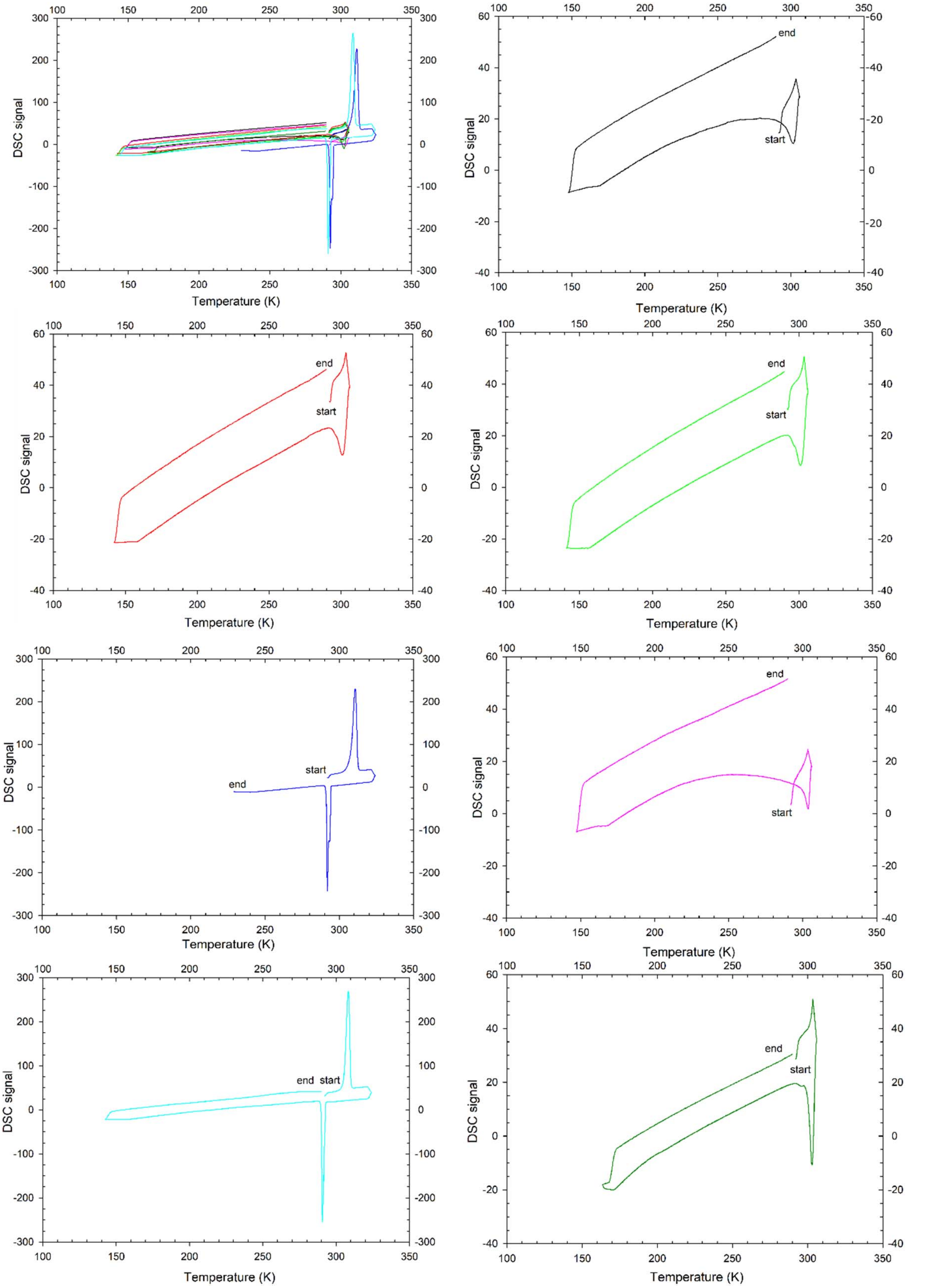

Figure S12A series of DSC cycles on *DL*-menthol heated (to below and above the melting point at 310 K). The DSC runs are grouped together in the first plot, and then shown separately.

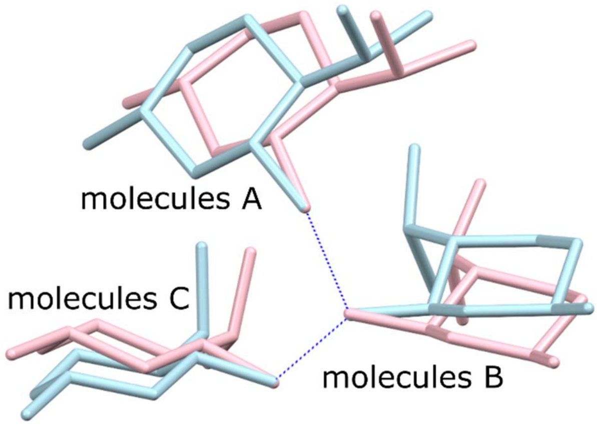

Figure S13 Chain intervals of the same enantiomorphic type molecules A, B and C, overlaid by strictly matching their O···O···O hydrogen bonds, in *DL*-menthol polymorphs α (blue) and β (pink).

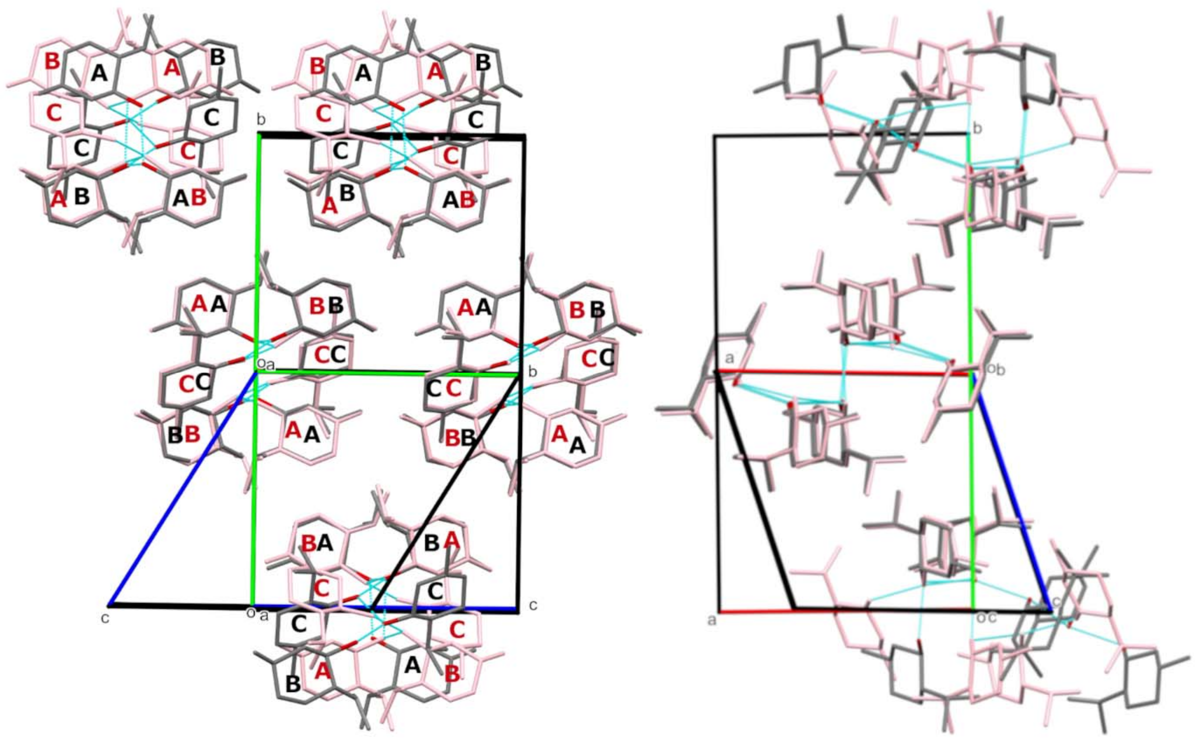

Figure S14 Superposition of the unit cells and structures of *DL*-menthol polymorphs α (grey) and β (pink).

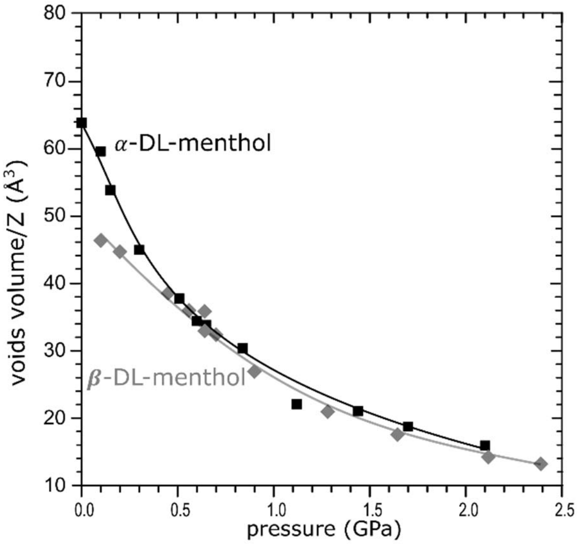

Figure S15 Voids volume per one menthol molecule (V_m=V/Z) as a function of pressure. Squares indicate α-*DL*-menthol and diamonds β-*DL*-menthol. The voids volume was calculated by program Mercury (Macrae *et al.*, 2020) for the probing-sphere radius of 0.6 Å and 0.1 Å steps.

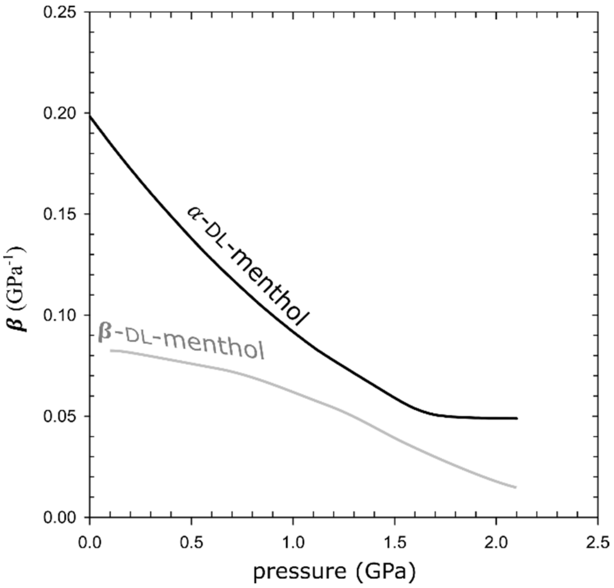

Figure S16 The compressibility coefficient $\beta=-(1/V_o)(\partial V/\partial p)$ for polymorphs α-*DL*-menthol and β-*DL*-menthol.

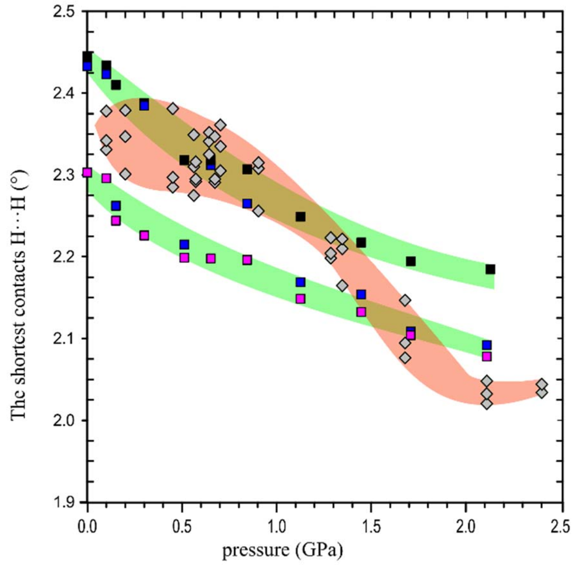

Figure S17 The shortest intermolecular contacts H···H of independent molecules A, B and C in the crystal structures of *DL*-menthol polymorph α (squares: pink for molecule A, blue for molecule B and black for molecule C) and β (grey diamonds).

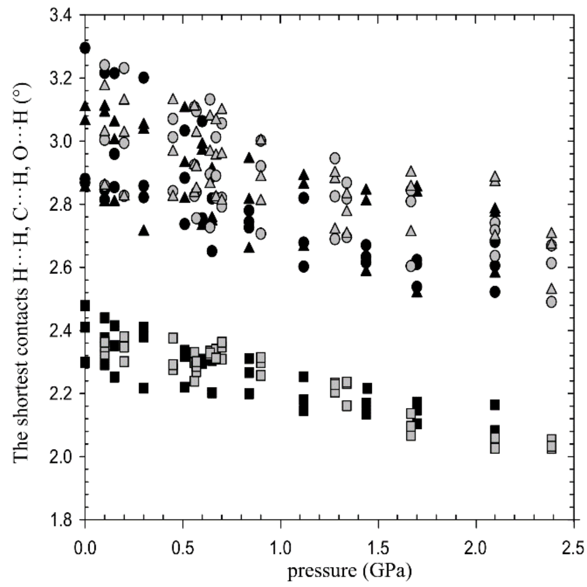

Figure S18 The shortest intermolecular distances H···H (squares), C···H (triangles) and non-H-bonding O···H (circles): black for polymorph α and gray for polymorph β.

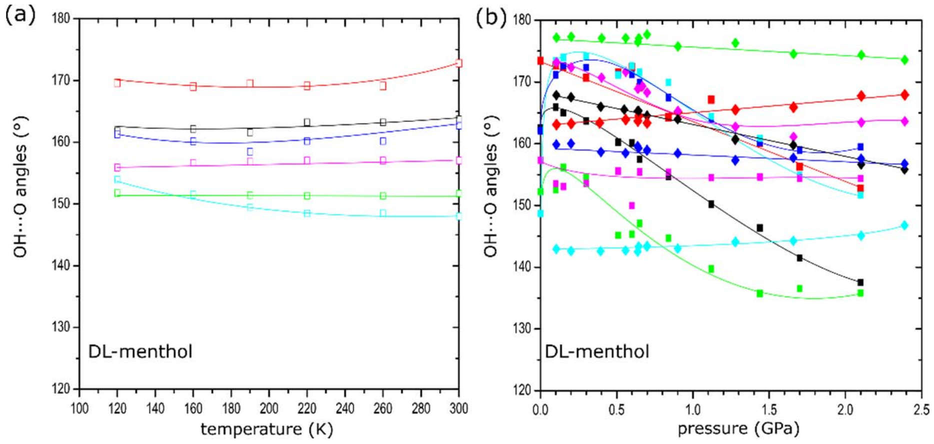

Figure S19(a) Temperature and (b) pressure dependence of OH···O angles in *DL*-menthol polymorphs α (squares) and β (diamonds). There are six independent OH···O angles, due to the disorder of protons: O1H1A···O1ii (green), O1H1B···O1B (pink), O1BH1BB···O1 (red), O1BH1BA···OC (cyan), O1CH1CA···O1B (blue) and OCH1C···O1Ci (black).

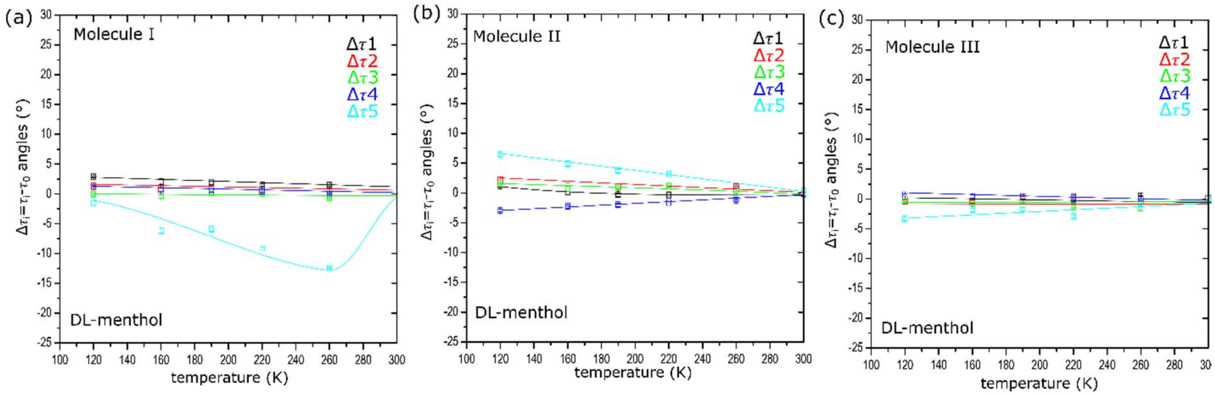

Figure S20 Changes in torsion angles τ_1 (black) τ_2 (red), τ_3 (green), τ_4 (blue) and τ_5 (cyan) in *DL*-menthol as a function of temperature. The molecule of menthol with its five most soft torsion angles, except those of the methyl and hydroxy rotors are shown in Figure S21c.

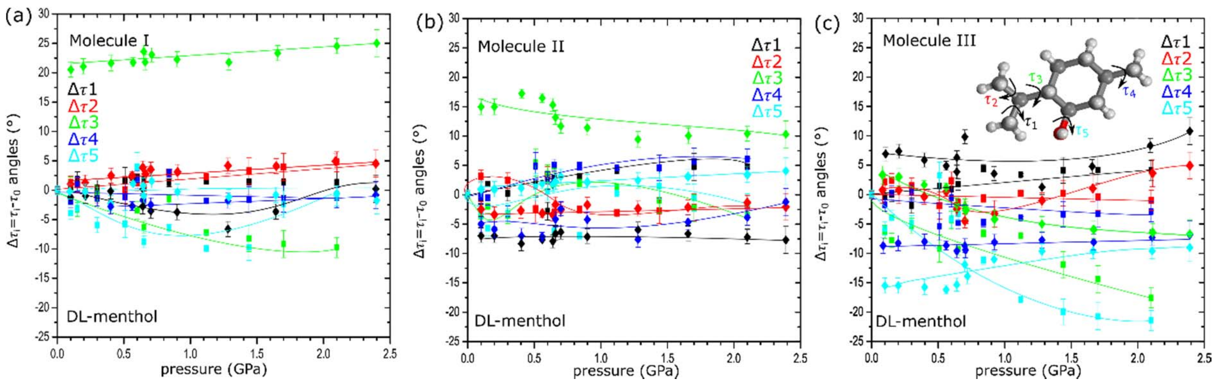

Figure S21 Changes in torsion angles (see the inset) τ_1 (black) τ_2 (red), τ_3 (green), τ_4 (blue) and τ_5 (cyan) in α-*DL*-menthol (squares) and β-*DL*-menthol (diamonds) as a function of pressure.

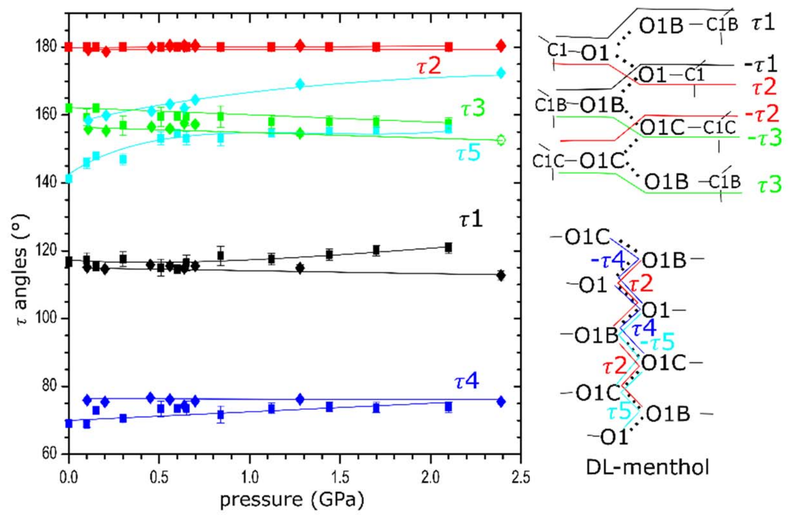

Figure S22 Changes in torsion angles C-O···O-C across the hydrogen bonds between molecules in one chain (see the scheme) τ_1 (black) τ_2 (red), τ_3 (green), τ_4 (blue) and τ_5 (cyan) in α-*DL*-menthol (squares) and β-*DL*-menthol (diamonds) as a function of pressure.

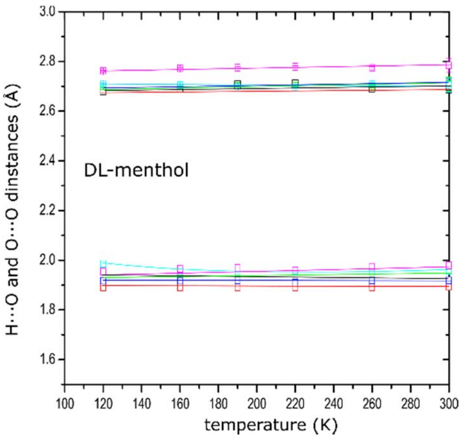

Figure S23 Temperature dependence of H-bonds: distances H···O (shorter) and O···O (longer) in α-*DL*-menthol. The code of colors is the same as in Figure 9.

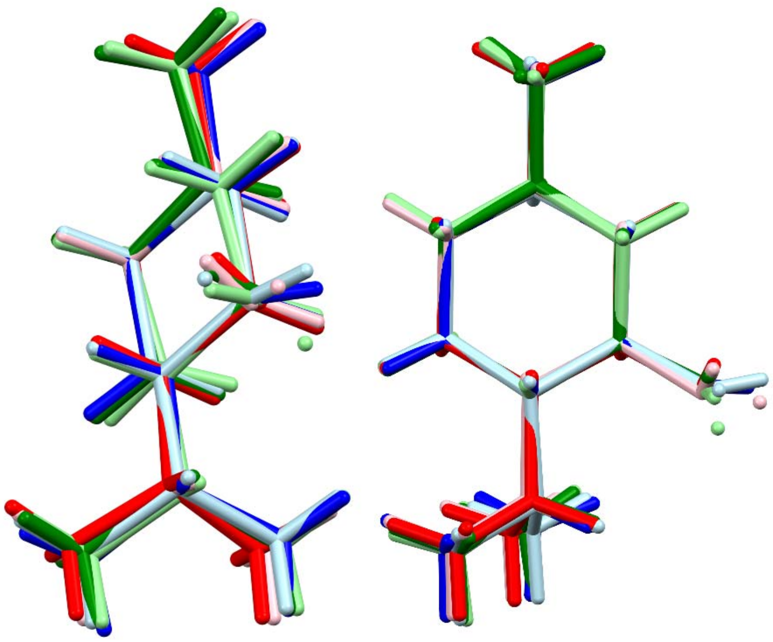

Figure S24 Molecules of *DL*-menthol overlaid with atoms C1, C4 and C4 possibly exactly superimposed. The applied colour code is: for α-*DL*-menthol light pink, light green and light blue; and for β-*DL*-menthol red, green and blue for molecules A, B and C, respectively. The left view is approximately along bonds O1-C1, C2-C3, C5-C6; and the right view is approximately perpendicular to the ring.

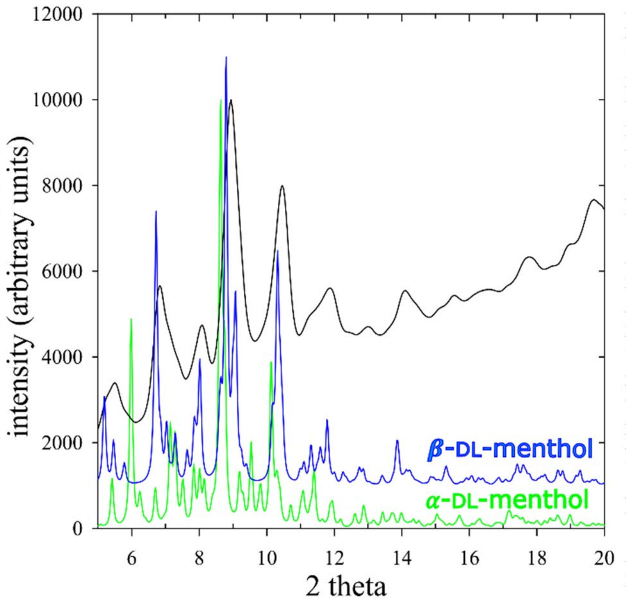

Figure S25 Powder X-ray diffraction (pXrd) pattern of the β-*DL*-menthol sample isothermally crystallized at 296 K from the *DL*-menthol solution in isopropanol (black line). The concentration of the

solution was so adjusted that the crystallization started at 0.63 GPa. The pXrd patterns calculated from single-crystal structures of polymorphs α-*DL*-menthol at 0.65 GPa (green) and β-*DL*-menthol at 0.64 GPa (blue) are shown for reference.

References

Cliffe, M.J. & Goodwin, A.L. (2012). PASCal: a principal axis strain calculator for thermal expansion and compressibility determination. *J. Appl. Cryst*. 45, 1321–1329. DOI: 10.1107/S0021889812043026

T. Langreiter & V. Kahlenberg (2015) TEV – a program for the determination and visualization of the thermal expansion tensor from diffraction data. *Crystals*, 5, 143–153 DOI: 10.3390/cryst5010143

Macrae, C. F., Sovago, I., Cottrell, S. J., Galek, P. T. A., McCabe, P., Pidcock, E., Platings, M., Shields, G. P., Stevens, J. S., Towler, M. & Wood, P. A. (2020). Mercury 4.0: from visualization to analysis, design and prediction. *J. Appl. Crystallogr.* **53**, 226–235. DOI: 10.1107/S1600576719014092
